# Vicinal difunctionalization of alkenes by four-component radical cascade reaction of xanthogenates, alkenes, CO, and sulfonyl oxime ethers

**DOI:** 10.3762/bjoc.15.176

**Published:** 2019-07-31

**Authors:** Shuhei Sumino, Takahide Fukuyama, Mika Sasano, Ilhyong Ryu, Antoine Jacquet, Frédéric Robert, Yannick Landais

**Affiliations:** 1 Department of Chemistry, Osaka Prefecture University, Sakai, Osaka 599-8531, Japan; 2 Department of Applied Chemistry, National Chiao Tung University, Hsinchu, Taiwan; 3 University of Bordeaux, Institute of Molecular Sciences, UMR-CNRS 5255, 351 cours de la libération, Talence, 33405 Cedex, France

**Keywords:** CO, multicomponent reaction, radicals, sulfonyl oxime ethers, xanthogenates

## Abstract

Four-component coupling reactions between xanthogenates, alkenes, CO, and sulfonyl oxime ethers were studied. In the presence of hexabutylditin, working as a propagating radical reagent, the chain reaction proceeds, as expected, taking into account reagents polarities, affording the corresponding functionalized α-keto oximes. Although yields are modest, this rare one-pot four-component process is easy to carry out and the resulting compounds, bearing multiple functionalities, have the potential for further elaboration.

## Introduction

**Scheme 1:**
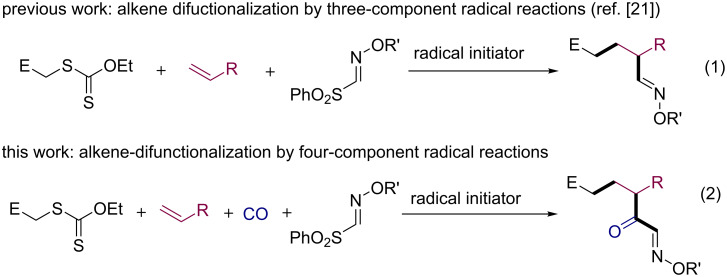
Concept: Alkene difuctionalization by four-component radical reaction using xanthates, alkenes, CO and sulfonyl oxime ethers.

Multicomponent reactions constitute a powerful and highly efficient tool in organic synthesis to build up intricate compounds from simple molecules in a single operation [1–5]. Needless to say, the contribution by radical chemistry is not trivial [5–7]. While alkenes and alkynes have served as efficient radical donor/acceptor type C2 synthons in multicomponent radical reactions, CO and isonitriles were shown to react as donor/acceptor type C1 synthons [6–15]. In this context, sulfonyl oxime ethers are powerful acceptors of type C1 synthon [8,16–17], which terminates the multicomponent reaction by a β-scission of RSO_2_ radicals [18–20]. Recently, one of us reported on a three-component radical reaction using xanthogenates, alkenes, and sulfonyl oxime ethers (Scheme 1, reaction 1) [21–22]. The reaction proceeds efficiently to provide good yields of α-alkoxyimino esters, potential precursors of lactams, lactones and β-keto esters. Since the three-component radical reaction involving alkyl halides and two radical C1 synthons, CO and sulfonyl oxime ethers, is known to be feasible [23–25], we were tempted to explore a novel class of four-component radical reaction [26–28] incorporating a xanthogenate, an alkene, CO, and a sulfonyl oxime ether (Scheme 1, reaction 2). This paper reports on the synthesis of functionalized α-keto oximes through such a one-pot, four-component procedure.

## Results and Discussion

We first investigated the reaction of xanthate **1a** [29], 1-octene (**2a**), CO, and sulfonyl oxime ether **3a** as a model reaction. When the mixture of **1a**, **2a** (5 equiv), and **3a** (1.2 equiv) in C_6_H_6_ (16 mL) in the presence of hexabutylditin as a radical mediator, and DTBHN (di-*tert*-butyl hyponitrite) as a radical initiator was heated under CO (130 atm) at 45 °C for 16 h, the envisaged four-component coupling product, keto oxime **5a**, was obtained in 43% yield, along with the three-component product **4a** (**4a**/**5a** = 9:91) (Table 1, entry 1). In this reaction, several unidentified byproducts were also formed. Since the conversion of **1a** (ca. 70%) was incomplete, a higher concentration ([**1a**] = 0.05 M) using 8 mL of C_6_H_6_ was employed, resulting in a higher conversion (ca. 80%), affording **5a** in 47% yield (Table 1, entry 2). The use of DCE (1,2-dichloroethane) as a solvent gave a 50% yield of **5a** (Table 1, entry 3). The present four-component product also proceeded under photoirradiation conditions in the absence of costly DTBHN (Table 1, entry 4). The reaction with 10 equivalents of **2a** together with 1.5 equivalents of **3a** led to a higher conversion (ca. 90%), affording acceptable yield and selectivity (Table 1, entry 5).

**Table 1:** Four-component coupling reaction of ethyl 2-((ethoxycarbonothioyl)thio)acetate (**1a**), 1-octene (**2a**), CO, and sulfonyl oxime ether **3a** under radical conditions^a^.

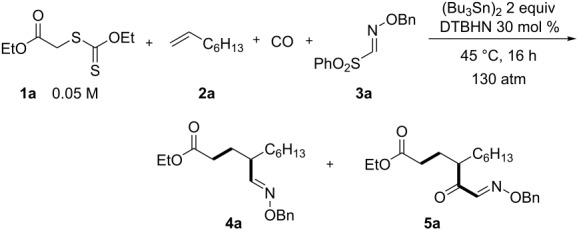					

entry	solvent	**2a** (equiv)	**3a** (equiv)	ratio^b^ (**4a**/**5a**)	**5a**^c^ (%)

1^d^	C_6_H_6_	5.0	1.2	9:91	43
2	C_6_H_6_	5.0	1.2	13:87	47 (39)
3	DCE	5.0	1.2	9:91	50 (43)
4^e^	DCE	5.0	1.2	8:92	41 (38)
5	DCE	10.0	1.5	13:87	56 (52)

^a^Reaction conditions: **1a** (0.4 mmol), **2a** (2 or 4 mmol), CO (130 atm), **3a** (0.48 or 0.6 mmol), DTBHN (0.12 mmol), (Bu_3_Sn)_2_ (0.8 mmol), C_6_H_6_ or DCE (8 mL), 45 °C, 16 h. ^b^Determined by GC. ^c^GC yields determined by using nonane as an internal standard. Isolated yields by silica gel chromatography are given in the parenthesis. ^d^C_6_H_6_ (16 mL). Conversion of **1a** = ca. 70%. ^e^Irradiation by Xe lamp was carried out in the absence of DTBHN.

**Figure 1:**
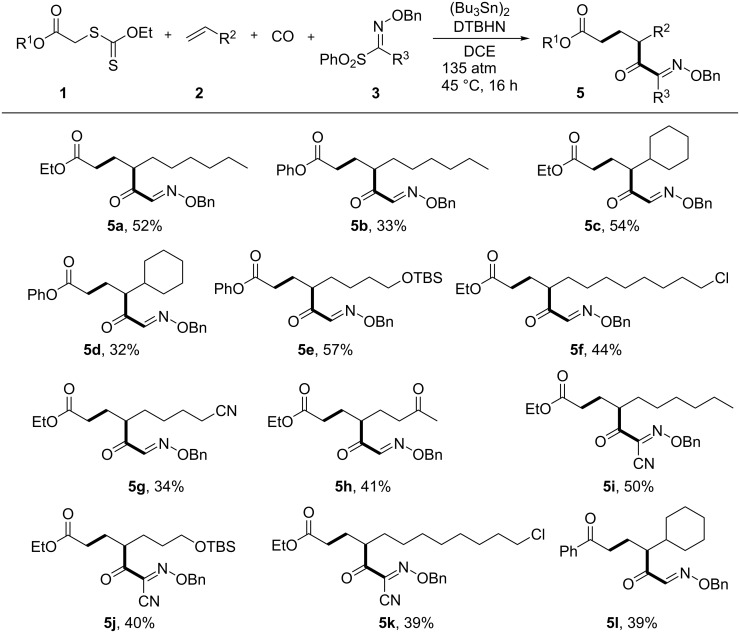
Vicinal difunctionalization of alkenes by four-component radical cascade reaction using xanthogenate **1**, alkenes **2**, CO, and sulfonyl oxime ethers **3** leading to **5a–l**. Reaction conditions: **1** (0.4 mmol), **2** (4 mmol), CO (130 atm), **3** (0.5 mmol), DTBHN (30 mol %), (Bu_3_Sn)_2_ (0.8 mmol), DCE (8 mL), 45 °C, 16 h.

With optimized reaction conditions in hand (Table 1, entry 5), we then examined the generality of this four-component radical cascade reaction using xanthates **1**, olefins **2**, CO, and oxime esters **3**, leading to **5a–l** (Figure 1). The xanthate **1b**, bearing a phenyl ester, gave similarly to **1a**, α-keto oxime **5b** in moderate yield. The reaction of **1a** or **1b** with vinylcyclohexane (**2b**) in the presence of CO and **3a** afforded the corresponding α-keto oximes **5c** and **5d** in 54 or 32% yield, respectively. The conditions were shown to be compatible with the presence of nitriles, ethers and halogens. Alkenes having a *tert*-butyldimethylsilyl ether such as 6-siloxy-1-hexene **2c** thus participated to the reaction to give **5e** in 57% yield. Alkenes having a chlorine atom, as in **2d**, were also competent substrates in the present four-component coupling reaction, affording **5f**, albeit in modest yield. The reaction of **1a** with 6-heptenenitrile (**2e**) and 5-hexen-2-one (**2f**) gave the corresponding four-component coupling products **5g** and **5h**, in 34 and 41% yield, respectively. The reaction with cyano-substituted sulfonyl oxime ester **3b** also worked well to provide cyano-functionalized α-keto oximes. **5i**, **5j**, and **5k** were thus accessible through the four-component coupling reaction between xanthogenates, alkenes, CO, and **3b** in acceptable isolated yields (39–50%). Finally, the reaction between acetophenone xanthate **1c**, **2b** and **3a** gave the corresponding keto oxime **5l** in 39% yield. The functionalized α-keto oximes obtained herein should be useful scaffolds for further functionalization. Indeed, the α-keto oximes were reported to be used for the synthesis of a variety of synthetic intermediates, including functionalized keto-aldehydes [22], aminoalcohols [30], triazoles [31], just to name a few.

**Figure 2:**
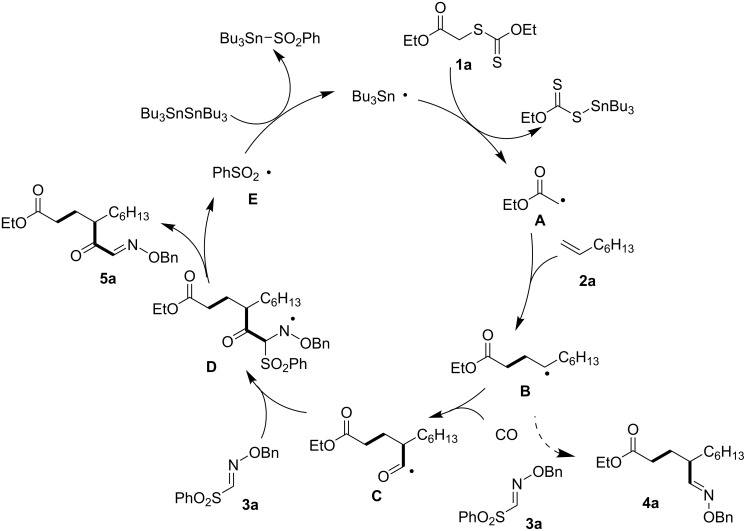
Proposed radical chain mechanism.

A reaction mechanism is finally proposed for the four-component cascade reaction, which is depicted in Figure 2 [22–24,32–34]. Initially, α-carbonyl radical **A** [8] was generated by the reaction of the tributyltin radical with **1a**. The electrophilic α-carbonyl radical **A** does not react with CO even at high CO pressure [6], and therefore selectively adds to electron-rich olefin **2a** to form a carbon-centered radical **B**. The radical **B**, regarded as a nucleophilic radical, then undergoes radical carbonylation with CO to give an acyl radical **C** [35], which then adds to electron-deficient sulfonyl oxime ether **3a** to afford **5a**. The resulting radical **D** then undergoes β-fragmentation providing **5a** along with the phenylsulfonyl radical **E**. S_H_2 reaction between radical **E** and hexabutylditin regenerates the tributyltin radical which sustains the radical chain. Since radical **B** can also add to sulfonyl oxime ether **3a**, we used high CO pressure conditions to encourage the radical carbonylation to form acyl radical **C**.

## Conclusion

In summary, we demonstrated that a four-component radical cascade reaction, between xanthogenates, alkenes, CO, and sulfonyl oxime ethers, can proceed under radical mediated conditions, using hexabutylditin as a radical chain carrier, to give the corresponding keto-oximes in moderate yields. A variety of functional groups are tolerated under the high CO pressure and temperature conditions. Among multicomponent reactions, specific four-component reactions are still rare [26–28]. The present procedure, which is easy to carry out using an autoclave in a single operation, shows that a fine tuning of the reaction conditions (pressure and temperature) and reagents polarities offer a straightforward access to polyfunctionalized substrates from readily available starting materials.

## Experimental

### General information

^1^H NMR spectra were recorded on a JEOL ECP-500 (500 MHz) and JEOL ECS-400 (400 MHz) spectrometers in CDCl_3_ and referenced at 0.00 ppm for TMS. ^13^C NMR spectra were recorded on a JEOL ECP-500 (125 MHz) and JEOL ECS-400 (100 MHz) spectrometers in CDCl_3_ and referenced at 77.00 ppm for CHCl_3_. Chemical shifts are reported in parts per million (δ). Splitting patterns are indicated as follows: br, broad; s, singlet; d, doublet; t, triplet; q, quartet; m, multiplet. Infrared spectra were obtained on a JASCO FT/IR-4100 spectrometer; absorptions were reported in reciprocal centimeters. High-resolution mass spectra were recorded on a JEOL MS700 spectrometer or Exactive Plus EMR (Thermo Fisher Scientific). The products were purified by flash chromatography on silica gel (Kanto Chem. Co. Silica Gel 60N (spherical, neutral, 40–50 μm)) and, if necessary, were further purified by recycling preparative HPLC (Japan Analytical Industry Co. Ltd., LC-918) equipped with GPC columns (JAIGEL-1H + JAIGEL-2H columns) using CHCl_3_ as eluent. Xanthogenate **1a**,**b** [20], **1c** [36], alkene **2c** [37], and oxime ester **3a**,**b** [20] were prepared according to reported procedures. Photoirradiation was carried out using a 500 W Xenon short arc lamp (Ushio Co. Ltd., lamp house: SX-UI500XQ, Xenon short arc lamp: UXL-500SX, power supply: BA-X500).

### Typical procedure for the synthesis of **5a** under thermal conditions

A magnetic stirring bar, **1a** (90.1 mg, 0.4 mmol), **2a** (455.3 mg, 4.0 mmol), **3a** (141.4 mg, 0.51 mmol), (Bu_3_Sn)_2_ (474.7 mg, 0.82 mmol), DTBHN (21.4 mg, 0.12 mmol), and 1,2-dichloroethane (8 mL) were placed in a stainless steel autoclave. The autoclave was closed, purged three times with carbon monoxide, pressurized with 130 atm of CO and then stirred at 45 °C for 16 h. Excess CO was discharged at room temperature after the reaction. The reaction mixture was then filtered and concentrated in vacuo to give a residue, which was subjected to silica gel column chromatography using hexane/EtOAc 10:1 as eluent, affording **5a** (83.7 mg, 0.22 mmol, 52%).

### Procedure for the synthesis of **5a** under photoirradiation conditions

A magnetic stirring bar, **1a** (82.0 mg, 0.39 mmol), **2a** (225.8 mg, 2.0 mmol), **3a** (133.1 mg, 0.48 mmol), (Bu_3_Sn)_2_ (452.6 mg, 0.78 mmol), and 1,2-dichloroethane (8 mL) were placed in a stainless-steel autoclave equipped with two sapphire glass windows and an inserted Pyrex glass liner. The autoclave was closed, purged three times with carbon monoxide, pressurized with 130 atm of CO and then irradiated by Xenon lamp (500 W) with stirring for 16 h. Excess CO was discharged after the reaction. The reaction mixture was then filtered and concentrated in vacuo to give a residue, which was subjected to silica gel column chromatography using hexane/EtOAc 10:1 as eluent, affording **5a** (52.9 mg, 0.15 mmol, 38%).

**Ethyl (*E*)-4-(2-((benzyloxy)imino)acetyl)decanoate (5a):** IR (neat, ZnSe) ν_max_ (cm^−1^): 3065, 2955, 1732, 1584, 1455, 1303, 1210; ^1^H NMR (CDCl_3_, 400 MHz) δ 7.49 (s, 1H), 7.38–7.33 (m, 5H), 5.25 (s, 2H), 4.10–4.08 (m, 2H), 3.35–3.33 (m, 1H), 2.21–2.17 (m, 2H), 1.25–1.21 (m, 11H), 0.87 (t, *J* = 6.8 Hz, 3H); ^13^C NMR (CDCl_3_, 100 MHz) δ 201.7, 173.0, 148.0, 136.1, 128.6, 128.6, 128.5, 77.9, 60.3, 45.3, 32.1, 32.0, 31.6, 29.2, 27.1, 26.4, 22.6, 14.2, 14.1; EIMS *m*/*z* (relative intensity): 316 (4), 227 (8), 199 (2), 91 (100); HRMS–EI (*m*/*z*): [M − C_2_H_5_O]^+^ calcd for C_19_H_26_NO_3_, 316.1913; found, 316.1916.

**Phenyl (*E*)-4-(2-((benzyloxy)imino)acetyl)decanoate (5b):** IR (neat, ZnSe) ν_max_ (cm^−1^): 2954, 2928, 1760, 1685, 1196, 1188; ^1^H NMR (CDCl_3_, 500 MHz) δ 7.54 (s, 1H), 7.39–7.34 (m, 8H), 7.24–7.21 (t, *J* = 7.4 Hz, 1H), 7.07–7.06 (d, *J* = 9.5 Hz, 11H), 5.25 (s, 2H), 3.49–3.44 (m, 1H), 2.57–2.45 (m, 2H), 2.13–2.04 (m, 1H), 1.98–1.89 (m, 1H), 1.72–1.62 (m, 1H), 1.48–1.40 (m, 1H), 1.29–1.22 (m, 10H), 0.89–0.86 (t, *J* = 7.5 Hz, 3H); ^13^C NMR (CDCl_3_, 125 MHz) δ 201.7, 171.7, 150.8, 148.2, 136.3, 129.5, 128.7, 128.6, 125.9, 121.7, 78.1, 45.4, 32.3, 32.2, 31.7, 29.4, 27.3, 26.5, 22.7, 14.2; EIMS *m*/*z* (relative intensity): 316 (1), 91 (100); HRMS–EI (*m*/*z*): [M − C_6_H_5_O]^+^ calcd for C_19_H_26_NO_3_, 316.1913; found, 316.1910.

**Ethyl (*E*)-6-((benzyloxy)imino)-4-cyclohexyl-5-oxohexanoate (5c):** IR (neat, ZnSe) ν_max_ (cm^−1^): 3065, 2978, 1681, 1497, 1370, 1251, 1210; ^1^H NMR (CDCl_3_, 500 MHz) δ 7,48 (s, 1H), 7.38–7.33 (m, 5H), 5.25 (s, 2H), 4.12–4.06 (m, 2H), 3.27–3.23 (m, 1H), 2.22–2.17 (m, 1H), 2,13–2.06 (m, 1H), 1.96–1.92 (m, 1H), 1.86–1.85 (m, 1H), 1.71–1.59 (m, 5H), 1.25–1.22 (t, *J* = 7 Hz, 3H), 1.17–0.90 (m, 5H); ^13^C NMR (CDCl_3_, 125 MHz) δ 201.1, 173.1, 148.7, 148.6, 136.2, 128.6, 128.5, 77.9, 60.3, 51.0, 50.9, 40.3, 32.2, 26.3, 23.4, 14.3, 14.1; EIMS *m*/*z* (relative intensity): 359 (1), 125 (2), 109 (6), 91 (100); HRMS–EI (*m*/*z*): [M]^+^ calcd for C_21_H_29_NO_4_, 359.2097; found, 359.2067.

**Phenyl (*E*)-6-((benzyloxy)imino)-4-cyclohexyl-5-oxohexanoate (5d):** IR (neat, ZnSe) ν_max_ (cm^−1^): 2927, 2852, 1759, 1682, 1492, 1197, 1135; ^1^H NMR (CDCl_3_, 500 MHz) δ 7.52 (s, 1H), 7.39–7.34 (m, 7H), 7.26–7.21 (m, 2H), 7.07–7.05 (m, 2H), 5.24 (s, 2H), 3.36–3.33 (m, 1H), 2.51–2.30 (m, 2H), 2.12–1.95 (m, 2H), 1.82–1.50 (m, 7H), 1.23–0.89 (m, 6H); ^13^C NMR (CDCl_3_, 100 MHz) δ 202.0, 171.8, 148.9, 136.4, 129.6, 128.9, 128.8, 128.8, 128.7, 126.0, 121.7, 78.1, 51.1, 40.5, 32.5, 31.6, 30.2, 26.5, 26.5, 23.5; EIMS *m*/*z* (relative intensity): 314 (53), 232 (8), 91 (100); HRMS–EI (*m*/*z*): [M − C_6_H_5_O]^+^ calcd for C_19_H_26_NO_3_, 314.1756, found, 314.1760.

**Phenyl (*E*)-4-(2-((benzyloxy)imino)acetyl)-8-((*tert*-butyldimethylsilyl)oxy)octanoate (5e):** IR (neat, ZnSe) ν_max_ (cm^−1^): 2952, 2854, 1760, 1686, 1595, 1493. ^1^H NMR (CDCl_3_, 500 MHz) δ 7.49 (s, 1H), 7.35–7.33 (m, 8H), 7.20–7.19 (m, 1H), 7.04–7.02 (m, 2H), 5.21 (s, 2H), 3.54–3.50 (m, 2H), 3.50–3.40 (m, 1H), 2.45–2.42 (m, 2H), 2.08–2.03 (m, 1H), 1.93–1.87 (m, 1H), 1.71–1.61 (m, 1H), 1.56–1.40 (m, 3H), 0.85 (s, 9H), 0.06 (s, 6H); ^13^C NMR (CDCl_3_, 125 MHz) δ 201.4, 171.5, 150.6, 148.0, 136.0, 129.4, 128.6, 125.8, 121.5, 78.6, 62.7, 44.9, 31.9, 30.3, 28.3, 26.7, 26.4, 25.9, 18.3, −5.1, −5.3; EIMS *m*/*z* (relative intensity): 440 (20), 404 (24), 263 (20), 91 (100); HRMS–EI (*m*/*z*): [M − OCH_2_Ph]^+^ calcd for C_22_H_34_NO_4_Si, 404.2257; found, 404.2260.

**Ethyl (*E*)-4-(2-((benzyloxy)imino)acetyl)-12-chlorododecanoate (5f):** IR (neat, ZnSe) ν_max_ (cm^−1^): 2930, 2856, 1731, 1682, 1455, 1371, 699; ^1^H NMR (CDCl_3_, 500 MHz) δ 7.49 (s, 1H), 7.38–7.34 (m, 5H), 5.25 (s, 2H), 4.12–4.07 (m, 2H), 3.54–3.51 (t, *J* = 6.5 Hz, 2H), 3.37–3.35 (m, 1H), 2.24–2.15 (m, 2H), 1.96–1.92 (m, 1H), 1.83–1.73 (m, 3H), 1.65–1.58 (m,1H), 1.42–1.37 (m, 3H), 1.25–1.22 (m, 11H); ^13^C NMR (CDCl_3_, 125 MHz) 201.6, 173.1, 148.1, 128.6, 128.5, 77.9, 60.3, 45.4, 45.1, 32.6, 32.1, 32.0, 29.5, 29.2, 28.8, 27.1, 26.8, 26.5, 14.2; EIMS *m*/*z* (relative intensity): 378 (1), 289 (3), 105 (1), 91 (100); HRMS–EI (*m*/*z*): [M − C_2_H_5_O]^+^ calcd for C_21_H_29_NO_3_Cl, 379.1419; found, 378.1842.

**Ethyl (*E*)-4-(2-((benzyloxy)imino)acetyl)-8-cyanooctanoate (5g):** IR (neat, ZnSe) ν_max_ (cm^−1^): 2938, 2246, 1683, 1731; ^1^H NMR (CDCl_3,_ 400 MHz) δ 7.50 (s, 1H), 7.35–7.41 (m, 5H), 5.26 (s, 2H), 4.08–4.14 (m, 2H), 3.34–3.40 (m, 1H), 2.14–2.27 (m, 5H), 1.90–1.97 (m, 1H), 1.77–1.82 (m, 1H), 1.54–1.68 (m, 3H), 1.29–1.47 (m, 2H), 1.24 (t, *J* = 7.6 Hz, 3H); ^13^C NMR (CDCl_3_, 100 MHz) δ 201.0, 172.8, 148.0, 136.0, 128.6, 128.5, 119.5, 77.9, 60.4, 44.7, 31.8, 31.0, 26.5, 26.2, 25.2, 16.9, 14.2; EIMS *m*/*z* (relative intensity): 313 (1), 91 (100), 77 (6), 55 (7); HRMS–EI (*m*/*z*): [M − C_2_H_5_O]^+^ calcd for C_18_H_21_N_2_O_3_, 313.1552; found, 313.1556.

**Ethyl (*E*)-4-(2-((benzyloxy)imino)acetyl)-7-oxooctanoate (5h):** IR (neat, ZnSe) ν_max_ (cm^−1^): 3510, 2936, 1732, 1715, 1684; ^1^H NMR (CDCl_3,_ 400 MHz) δ 7.49 (s, 1H), 7.38–7.26 (m, 5H), 5.25 (s, 2H), 4.12–4.07 (m, 2H), 3.39–3.35 (m, 1H), 2.40–2.27 (m, 2H), 2.27–2.20 (m, 2H), 2.06 (s, 3H), 2.00–1.85 (m, 2H), 1.82–1.73 (m, 2H), 1.25–1.22 (t, *J* = 7.2 Hz, 3H); ^13^C NMR (CDCl_3,_ 100 MHz,) δ 207.7, 200.9, 172.9, 147.9, 136.0, 128.6, 128.6, 128.5, 78.0, 60.4, 44.3, 40.6, 31.7, 29.9, 26.5, 25.2, 14.2; EIMS *m*/*z* (relative intensity): 302 (2), 91 (100), HRMS–EI (*m*/*z*): [M − C_2_H_5_O]^+^ calcd for C_17_H_20_NO_4_, 302.1392; found, 302.1390.

**Ethyl (*E*)-4-(2-((benzyloxy)imino)-2-cyanoacetyl)decanoate (5i):** IR (neat, ZnSe) ν_max_ (cm^−1^): 2930, 2857, 1733, 1698, 1455, 1035; ^1^H NMR (CDCl_3_, 500 MHz) δ 7.43–7.38 (m, 5H), 5.50 (s, 2H), 4.12–4.06 (m, 2H), 3.37–3.32 (m, 1H), 2.26–2.14 (m, 2H), 2.01–1.94 (m, 1H), 1.85–1.80 (m, 1H), 1.66–1.60 (m, 1H), 1.44–1.33 (m, 1H), 1.28–1.19 (m, 11H), 0.89–0.86 (t, *J* = 6.5 Hz, 3H); ^13^C NMR (CDCl_3_, 125 MHz) δ 195.4, 172.7, 134.4, 132.4, 129.2, 128.9, 128.8, 107.4, 80.6, 60.4, 45.4, 31.9 31.6, 31.5, 29.1, 27.0, 26.1, 22.5, 14.2, 14.1; EIMS *m*/*z* (relative intensity): 341 (2), 200 (6), 131 (8), 91 (100); HRMS–EI (*m*/*z*): [M − C_2_H_5_O]^+^ calcd for C_20_H_26_N_2_O_3_, 341.1865; found, 341.1867.

**Ethyl (*E*)-4-(2-((benzyloxy)imino)-2-cyanoacetyl)-7-((*tert*-butyldimethylsilyl)oxy)heptanoate (5j):** IR (neat, ZnSe) ν_max_ (cm^−1^): 2929, 2857, 1732, 1698, 1255; ^1^H NMR (CDCl_3_, 500 MHz) δ 7.34 (m, 5H), 5.47 (s, 2H), 4.12–4.05 (m, 2H), 3.53–3.48 (m, 2H), 3.39–3.36 (m, 1H), 2.28–2.18 (m, 2H), 2.06–1.93 (m, 1H), 1.85–1.78 (m, 1H), 1.72–1.61 (m, 1H), 1.59–1.50 (m, 1H), 1.41–1.38 (m, 2H), 1.26–1.21 (t, 3H), 0.88 (s, 9H), 0.02 (s, 6H); ^13^C NMR (CDCl_3_, 125 MHz) δ 195.2, 172.6, 134.3, 132.4, 129.2, 129.0, 128.8, 107.3, 80.7, 62.5, 60.5, 41.1, 31.5, 30.1, 28.0, 26.2, 25.9, 18.3, 14.2, −5.2; EIMS *m*/*z* (relative intensity): 417 (13), 215 (4), 131 (5), 91 (100); HRMS–EI (*m*/*z*): [M − C_4_H_9_]^+^ calcd for C_21_H_29_N_2_O_5_Si, 417.1846; found, 417.1853.

**Ethyl (*E*)-4-(2-((benzyloxy)imino)-2-cyanoacetyl)-12-chlorododecanoate (5k):** IR (neat, ZnSe) ν_max_ (cm^−1^): 2932, 2857, 1731, 1698, 1035; ^1^H NMR (CDCl_3_, 500 MHz) δ 7.40 (m, 5H), 5.48 (s, 2H), 4.12–4.07 (m, 2H), 3.55–3.53 (t, *J* = 6.5 Hz, 2H) 3.36–3.33 (m, 1H), 2.29–2.18 (m, 2H), 2.00–1.92 (m, 1H), 1.85–1.74 (m, 3H), 1.67–1.62 (m, 1H), 1.42–1.38 (m, 3H), 1.25–1.12 (m, 12H); ^13^C NMR (CDCl_3_, 125 MHz) δ 195.4, 172.7, 134.3, 132.4, 129.2, 129.0, 128.8, 107.3, 80.6, 60.4, 45.4, 45.1, 32.5, 31.8, 31.6, 29.3, 29.1, 28.7, 27.0, 26.7, 26.1, 14.2; EIMS *m*/*z* (relative intensity): 404 (1), 181 (16), 169 (14), 131 (23), 119 (17), 91 (100); HRMS–EI (*m*/*z*): [M − C_2_H_5_O]^+^ calcd for C_22_H_29_N_2_O_3_Cl, 404.1867; found, 404.1858.

**(*E*)-3-Cyclohexyl-2,6-dioxo-6-phenylhexanal *O*-benzyl oxime (5l):** IR (neat, ZnSe) ν_max_ (cm^−1^): 2926, 2852, 1682, 1449, 1208; ^1^H NMR (CDCl_3_, 400 MHz) δ 7.89–7.88 (m, 2H), 7.54–7.32 (m, 8H), 5.17 (s, 2H), 3.34–3.30 (m, 1H), 2.89–2.69 (m, 2H), 2.02–2.00 (m, 2H), 1.69–1.51 (m, 6H), 1.16–0.88 (m, 6H); ^13^C NMR (CDCl_3_, 100 MHz) 202.3, 199.5, 148.6, 136.7, 136.1, 132.9, 128.7, 128.5, 128.5, 128.4, 128.0, 77.8, 51.1, 40.2, 36.1, 31.4, 29.9, 26.3, 22.6; EIMS *m*/*z* (relative intensity): 300 (6), 284 (9), 257 (5), 91 (100); HRMS–EI (*m*/*z*): [M − OCH_2_Ph]^+^ calcd for C_18_H_22_NO_2_, 284.1651; found, 284.1645.

## Supporting Information

File 1 Copies of NMR spectra.
