# Supplementary material for: Vicinal difunctionalization of alkenes by four-component radical cascade reaction of xanthogenates, alkenes, CO, and sulfonyl oxime ethers
Source: Beilstein J Org Chem. 2019 Jul 31;15:1822–8. doi: 10.3762/bjoc.15.176 (PMC6693370; doi:10.3762/bjoc.15.176)
Supplement: File 1 — Copies of NMR spectra. [file Beilstein_J_Org_Chem-15-1822-s001.pdf]

BEILSTEIN JOURNAL OF ORGANIC CHEMISTRY

Supporting Information

for

Vicinal difunctionalization of alkenes by four-component radical cascade reaction of xanthogenates, alkenes, CO, and sulfonyl oxime ethers

Shuhei Sumino, Takahide Fukuyama, Mika Sasano, Ilhyong Ryu, Antoine Jacquet, Frédéric Robert and Yannick Landais

Beilstein J. Org. Chem. **2019,** *15,* 1822–1828. doi:10.3762/bjoc.15.176

Copies of NMR spectra

License and Terms: This is a supporting information file under the terms of the Creative Commons Attribution License (http://creativecommons.org/licenses/by/4.0). Please note that the reuse, redistribution and reproduction in particular requires that the authors and source are credited.
The license is subject to the *Beilstein Journal of Organic Chemistry* terms and conditions: (https://www.beilstein-journals.org/bjoc)

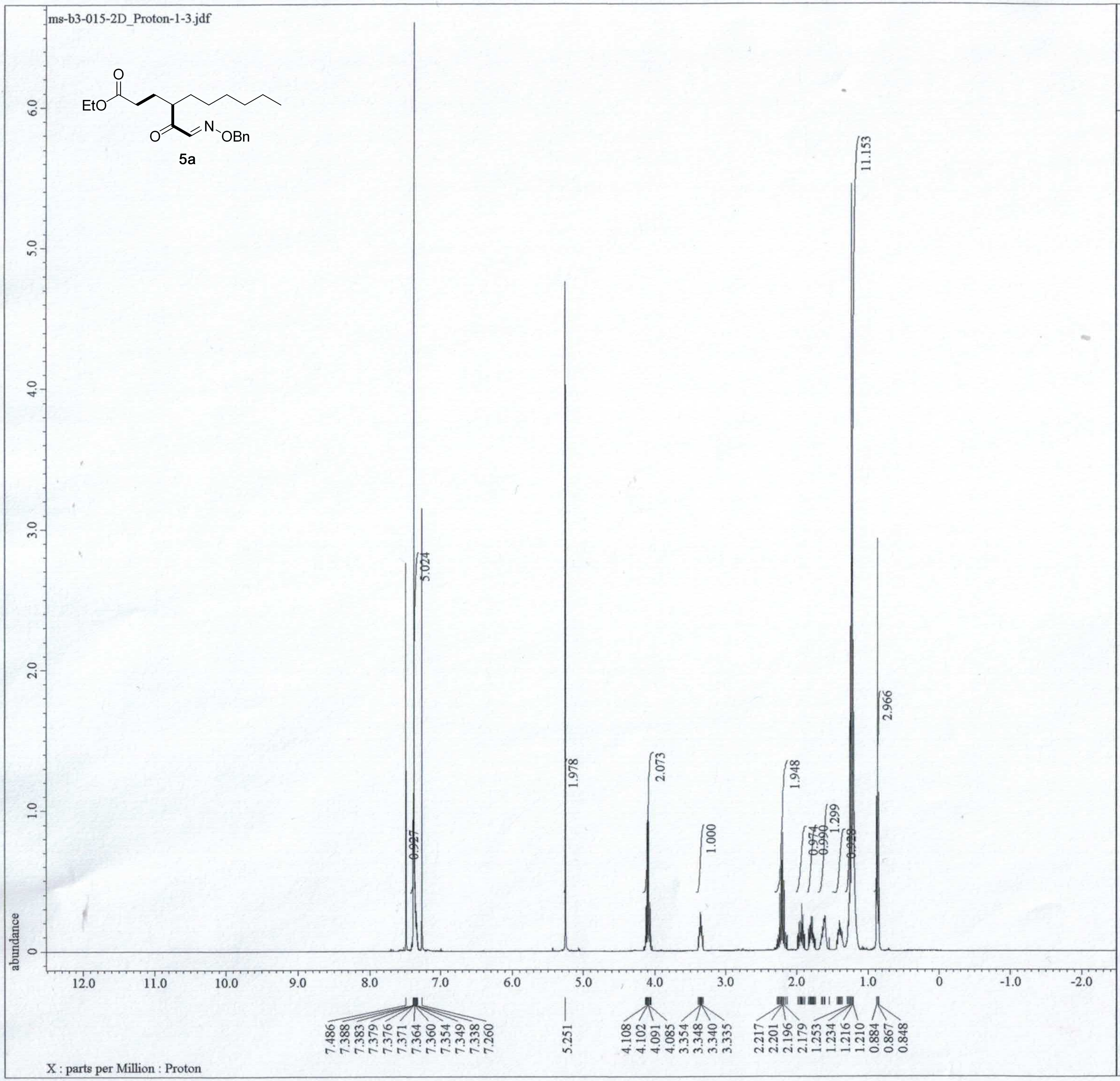

JEOL RESONANCE

Filename	= ms-b3-015-2D_Proton-1-3.jd
Author	= delta
Experiment	= proton.jxp
Sample_Id	= ms-b3-015-2D
Solvent	= CHLOROFORM-D
Creation_Time	= 26-DEC-2013 00:17:06
Revision_Time	= 26-DEC-2013 14:13:22
Current_Time	= 26-DEC-2013 14:13:28
Comment	= single_pulse
Data_Format	= 1D COMPLEX
Dim_Size	= 13107
Dim_Title	= Proton
Dim_Units	= [ppm]
Dimensions	= X
Site	= JNM-ECS400
Spectrometer	= DELTA2_NMR
Field_Strength	= 9.42499681[T] (400[MHz])
X_Acq_Duration	= 2.1757952[s]
X_Domain	= 1H
X_Freq	= 401.28219856[MHz]
X_Offset	= 5[ppm]
X_Points	= 16384
X_Prescans	= 1
X_Resolution	= 0.45960208[Hz]
X_Sweep	= 7.53012048[kHz]
X_Sweep_Clipped	= 6.02409639[kHz]
Irr_Domain	= Proton
Irr_Freq	= 401.28219856[MHz]
Irr_Offset	= 5[ppm]
Tri_Domain	= Proton
Tri_Freq	= 401.28219856[MHz]
Tri_Offset	= 5[ppm]
Clipped	= FALSE
Scans	= 32
Total_Scans	= 32
Relaxation_Delay	= 5[s]
Recvr_Gain	= 40
Temp_Get	= 20.4[dC]
X_90_Width	= 9.25[us]
X_Acq_Time	= 2.1757952[s]
X_Angle	= 45[deg]
X_Atn	= 0.8[dB]
X_Pulse	= 4.625[us]
Irr_Mode	= Off
Tri_Mode	= Off
Dante_Presat	= FALSE
Initial_Wait	= 1[s]
Repetition_Time	= 7.1757952[s]

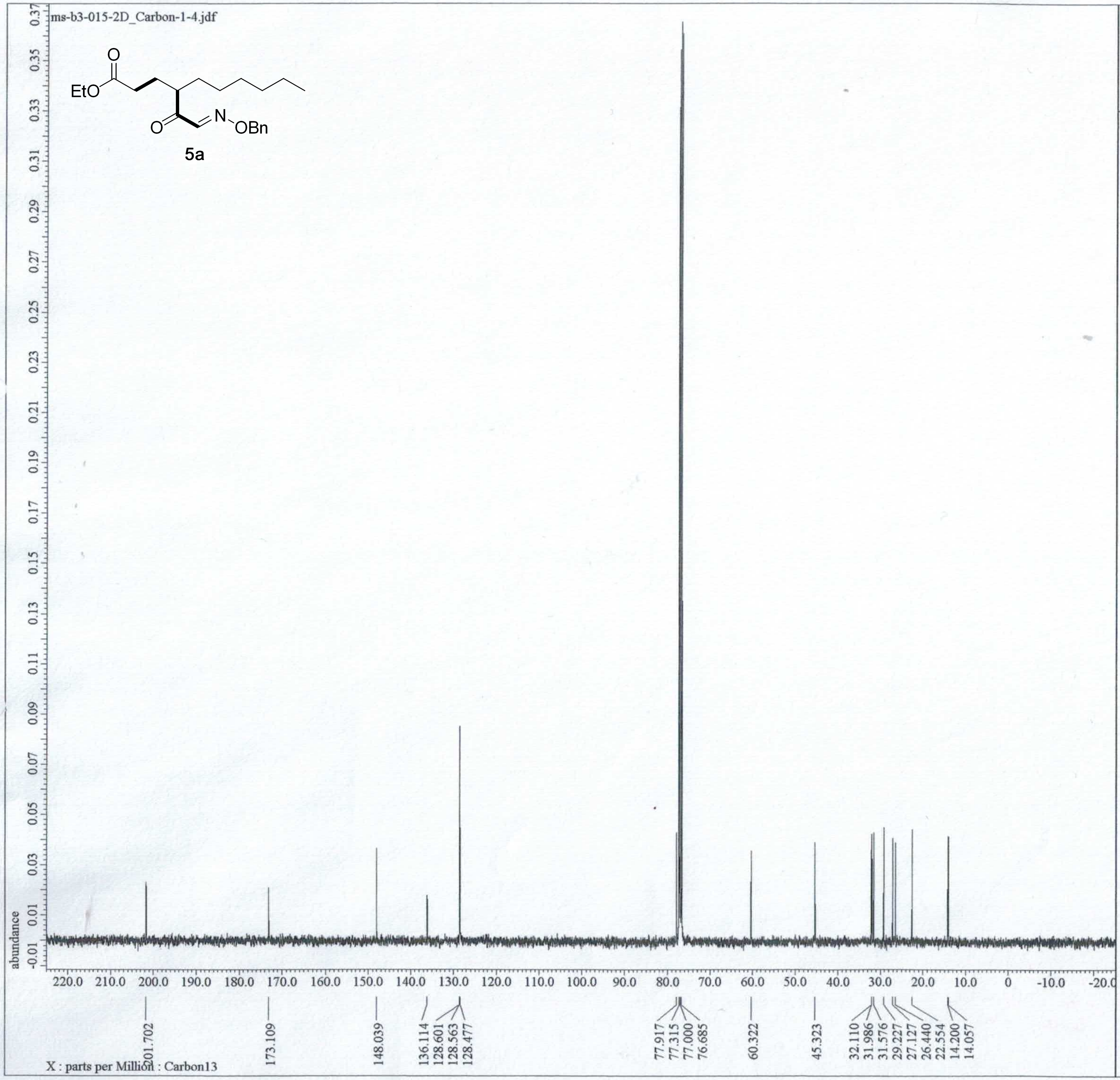

JEOL RESONANCE

Filename	= ms-b3-015-2D_Carbon-1-4.jd
Author	= delta
Experiment	= carbon.jxp
Sample_Id	= ms-b3-015-2D
Solvent	= CHLOROFORM-D
Creation_Time	= 26-DEC-2013 02:13:45
Revision_Time	= 26-DEC-2013 14:14:15
Current_Time	= 26-DEC-2013 14:14:25
Comment	= single pulse decoupled gat
Data_Format	= 1D COMPLEX
Dim_Size	= 26214
Dim_Title	= Carbon13
Dim_Units	= [ppm]
Dimensions	= X
Site	= JNM-ECS400
Spectrometer	= DELTA2_NMR
Field_Strength	= 9.42499681[T] (400[MHz])
X_Acq_Duration	= 1.03809024[s]
X_Domain	= 13C
X_Freq	= 100.90247863[MHz]
X_Offset	= 100[ppm]
X_Points	= 32768
X_Prescans	= 4
X_Resolution	= 0.96330739[Hz]
X_Sweep	= 31.56565657[kHz]
X_Sweep_Clipped	= 25.25252525[kHz]
Irr_Domain	= Proton
Irr_Freq	= 401.28219856[MHz]
Irr_Offset	= 5[ppm]
Clipped	= FALSE
Scans	= 512
Total_Scans	= 512
Relaxation_Delay	= 2[s]
Recvr_Gain	= 50
Temp_Get	= 20.8[dC]
X_90_Width	= 8.75[us]
X_Acq_Time	= 1.03809024[s]
X_Angle	= 30[deg]
X_Atn	= 5.2[dB]
X_Pulse	= 2.91666667[us]
Irr_Atn_Dec	= 22.691[dB]
Irr_Atn_Noe	= 22.691[dB]
Irr_Noise	= WALTZ
Irr_Pwidth	= 0.115[ms]
Decoupling	= TRUE
Initial_Wait	= 1[s]
Noe	= TRUE
Noe_Time	= 2[s]
Repetition_Time	= 3.03809024[s]

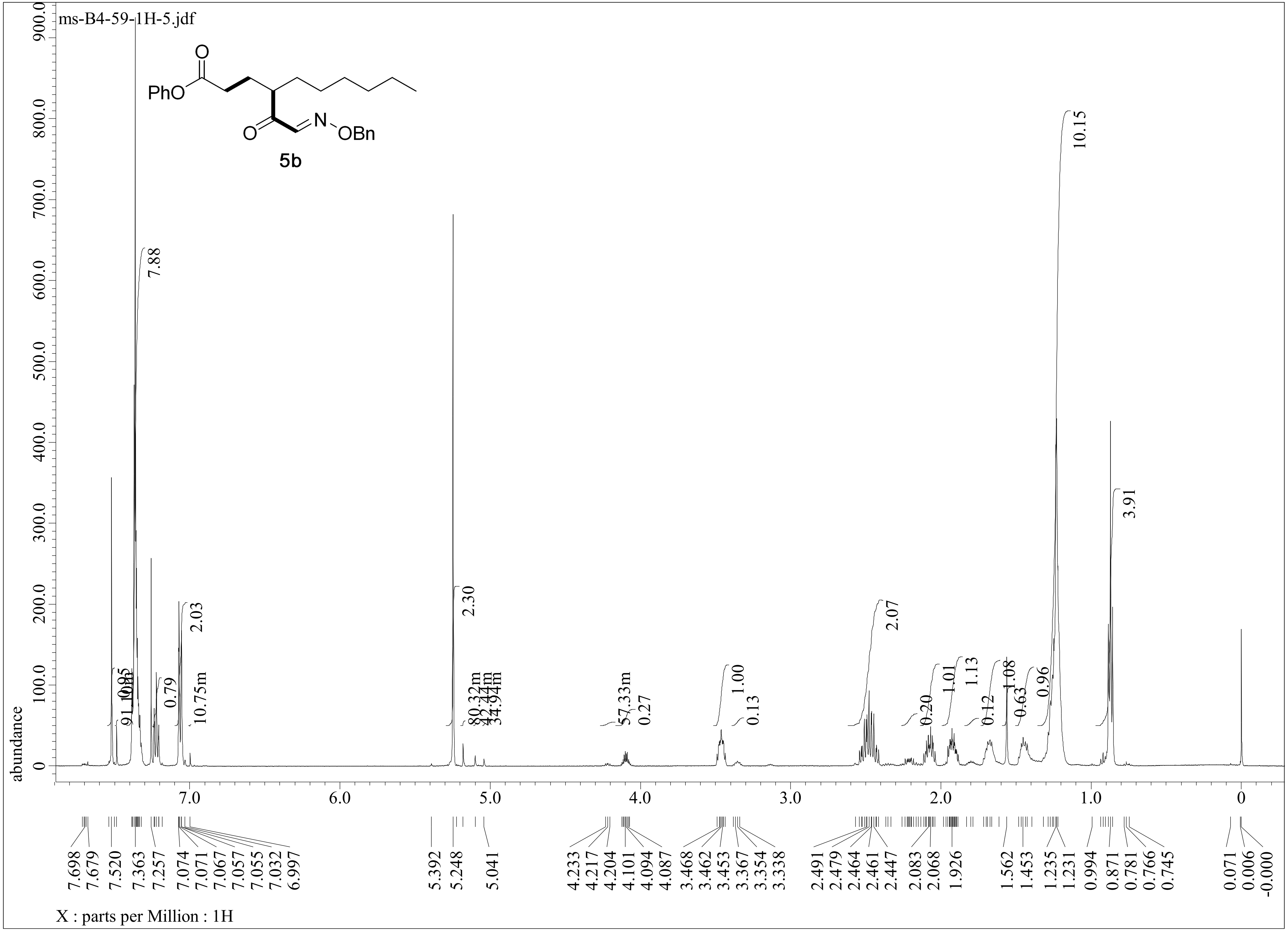
ms-B4-59-1H-5.jdf
PhO
O
O
N
OBn
5b
abundance
X : parts per Million : 1H
7.88
2.30
2.03
10.75m
0.79
2.07
1.00
0.13
0.27
57.33m
1.01
1.13
1.08
0.96
0.63
0.12
0.20
10.15
3.91
7.698 7.679 7.520 7.363 7.257 7.074 7.071 7.067 7.057 7.055 7.032 6.997
5.392 5.248 5.041
4.233 4.217 4.204 4.101 4.094 4.087 3.468 3.462 3.453 3.367 3.354 3.338
2.491 2.479 2.464 2.461 2.447 2.083 2.068 1.926
1.562 1.453 1.235 1.231 0.994 0.871 0.781 0.766 0.745
0.071 0.006 -0.000

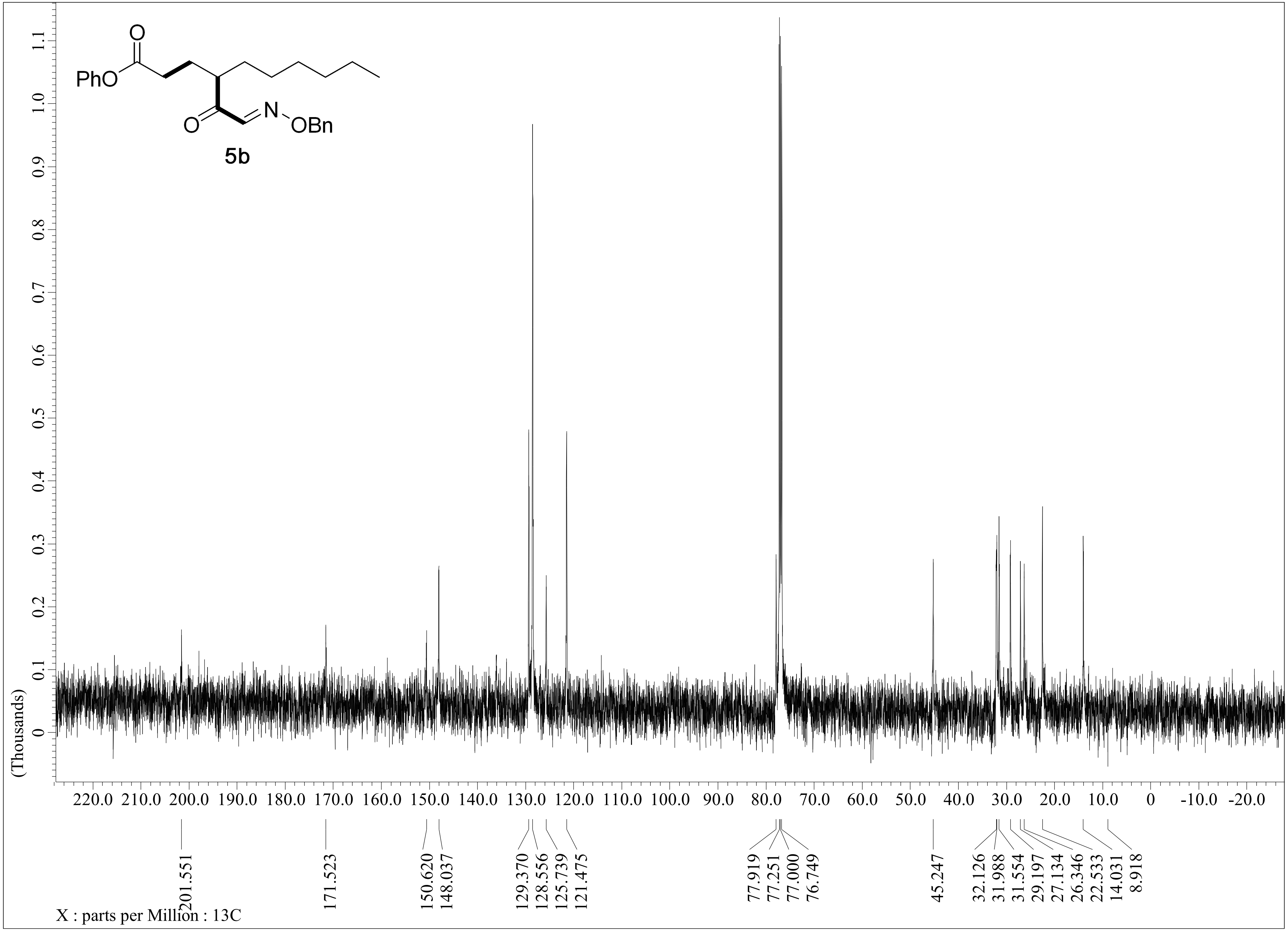
PhO
O
O
N
OBn
5b
(Thousands)
X : parts per Million : 13C
201.551
171.523
150.620
148.037
129.370
128.556
125.739
121.475
77.919
77.251
77.000
76.749
45.247
32.126
31.988
31.554
29.197
27.134
26.346
22.533
14.031
8.918

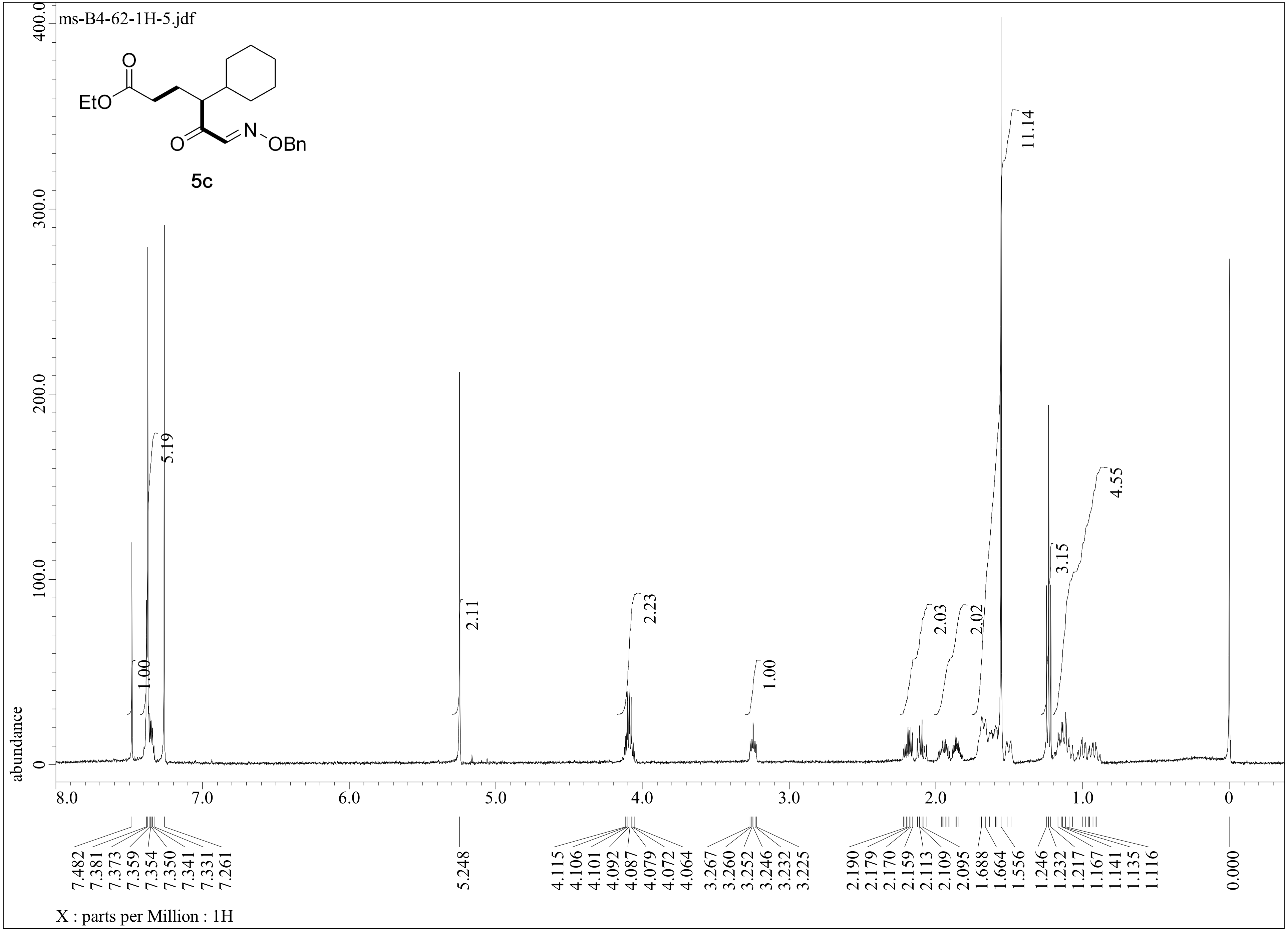
ms-B4-62-1H-5.jdf
EtO
O
N
OBn
5c
abundance
400.0
300.0
200.0
100.0
0
5.19
1.00
2.11
2.23
1.00
2.03
2.02
11.14
3.15
4.55
8.0
7.0
6.0
5.0
4.0
3.0
2.0
1.0
0
7.482 7.381 7.373 7.359 7.354 7.350 7.341 7.331 7.261
5.248
4.115 4.106 4.101 4.092 4.087 4.079 4.072 4.064
3.267 3.260 3.252 3.246 3.232 3.225
2.190 2.179 2.170 2.159 2.113 2.109 2.095
1.688 1.664 1.556
1.246 1.232 1.217 1.167 1.141 1.135 1.116
0.000
X : parts per Million : 1H

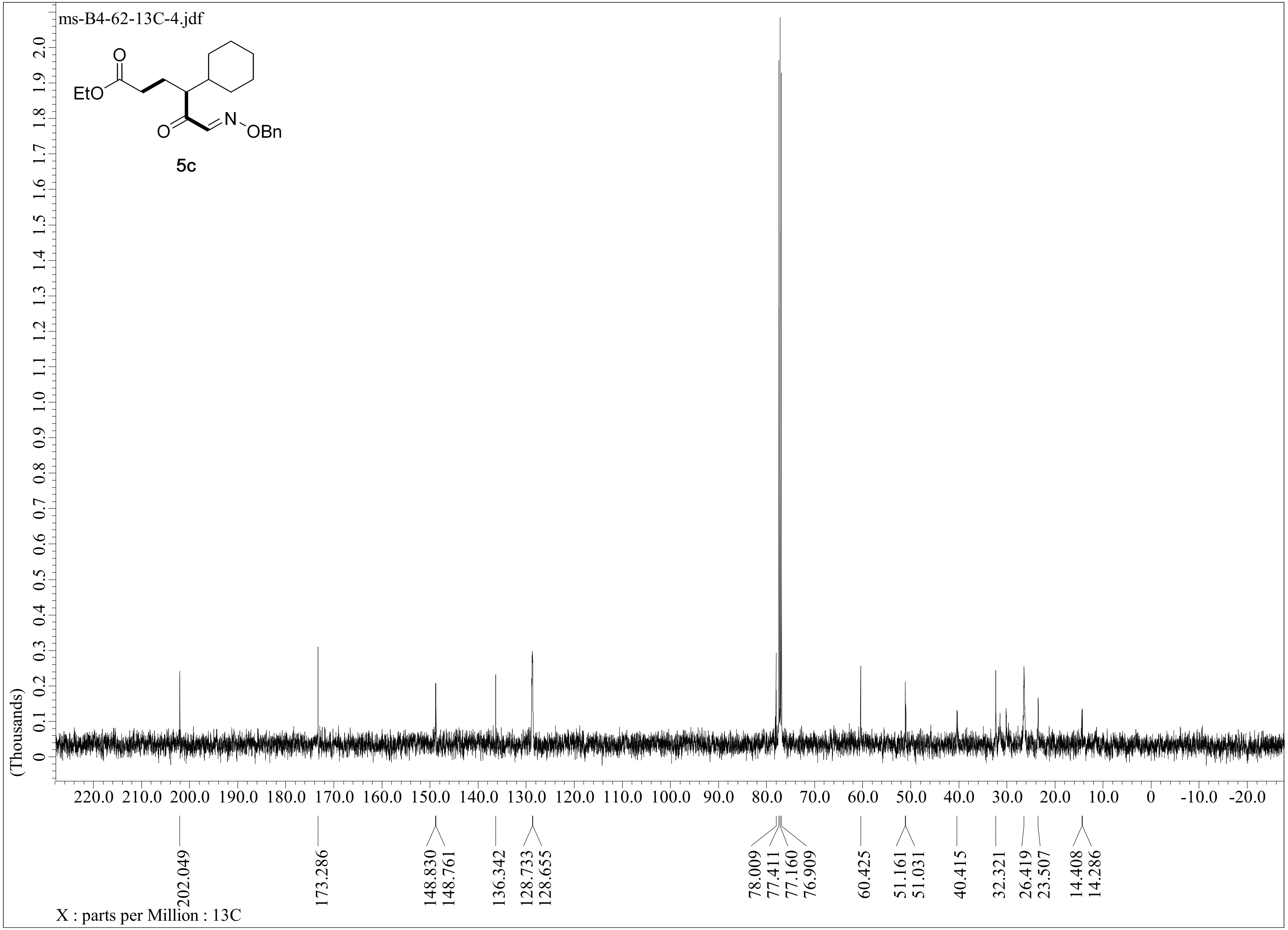
ms-B4-62-13C-4.jdf
EtO
O
O
N
OBn
5c
(Thousands)
X : parts per Million : 13C
202.049
173.286
148.830
148.761
136.342
128.733
128.655
78.009
77.411
77.160
76.909
60.425
51.161
51.031
40.415
32.321
26.419
23.507
14.408
14.286

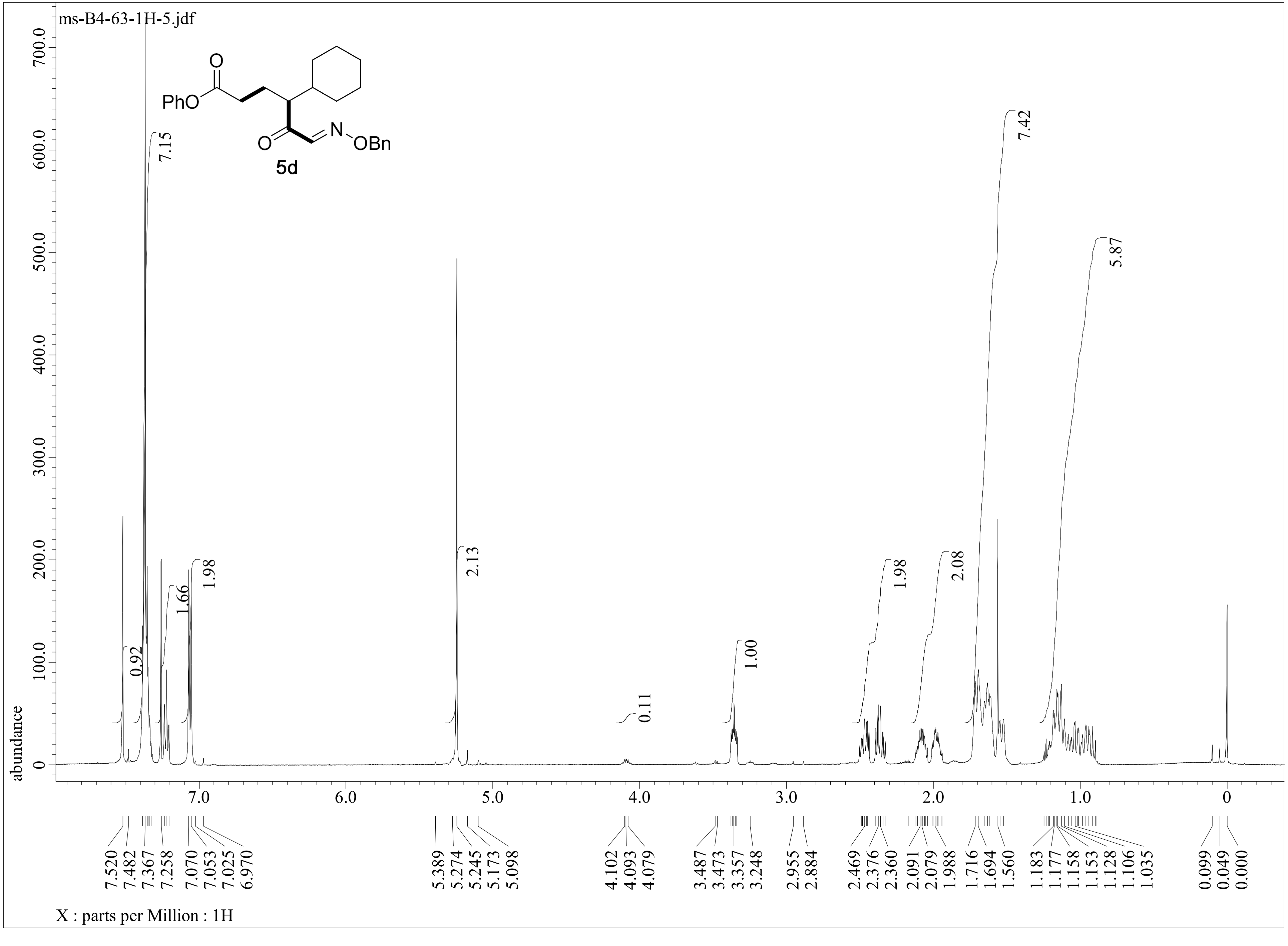
ms-B4-63-1H-5.jdf
PhO
O
N
OBn
5d
abundance
700.0
600.0
500.0
400.0
300.0
200.0
100.0
0
7.15
0.92
1.66
1.98
2.13
0.11
1.00
1.98
2.08
7.42
5.87
7.0
6.0
5.0
4.0
3.0
2.0
1.0
0
7.520
7.482
7.367
7.258
7.070
7.053
7.025
6.970
5.389
5.274
5.245
5.173
5.098
4.102
4.093
4.079
3.487
3.473
3.357
3.248
2.955
2.884
2.469
2.376
2.360
2.091
2.079
1.988
1.716
1.694
1.560
1.183
1.177
1.158
1.153
1.128
1.106
1.035
0.099
0.049
0.000
X : parts per Million : 1H

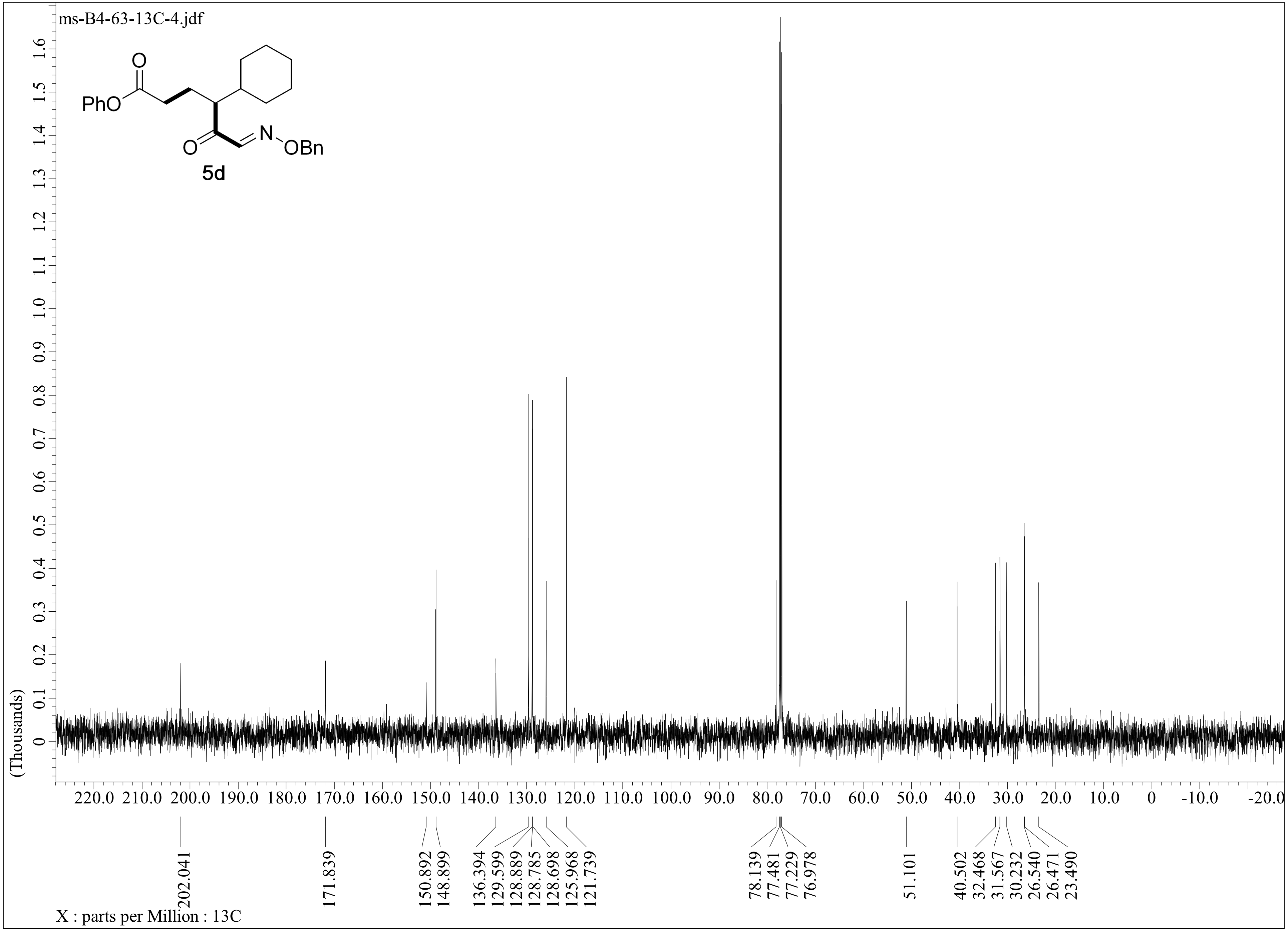
ms-B4-63-13C-4.jdf
PhO
O
N
OBn
O
5d
(Thousands)
X : parts per Million : 13C
202.041
171.839
150.892
148.899
136.394
129.599
128.889
128.785
128.698
125.968
121.739
78.139
77.481
77.229
76.978
51.101
40.502
32.468
31.567
30.232
26.540
26.471
23.490

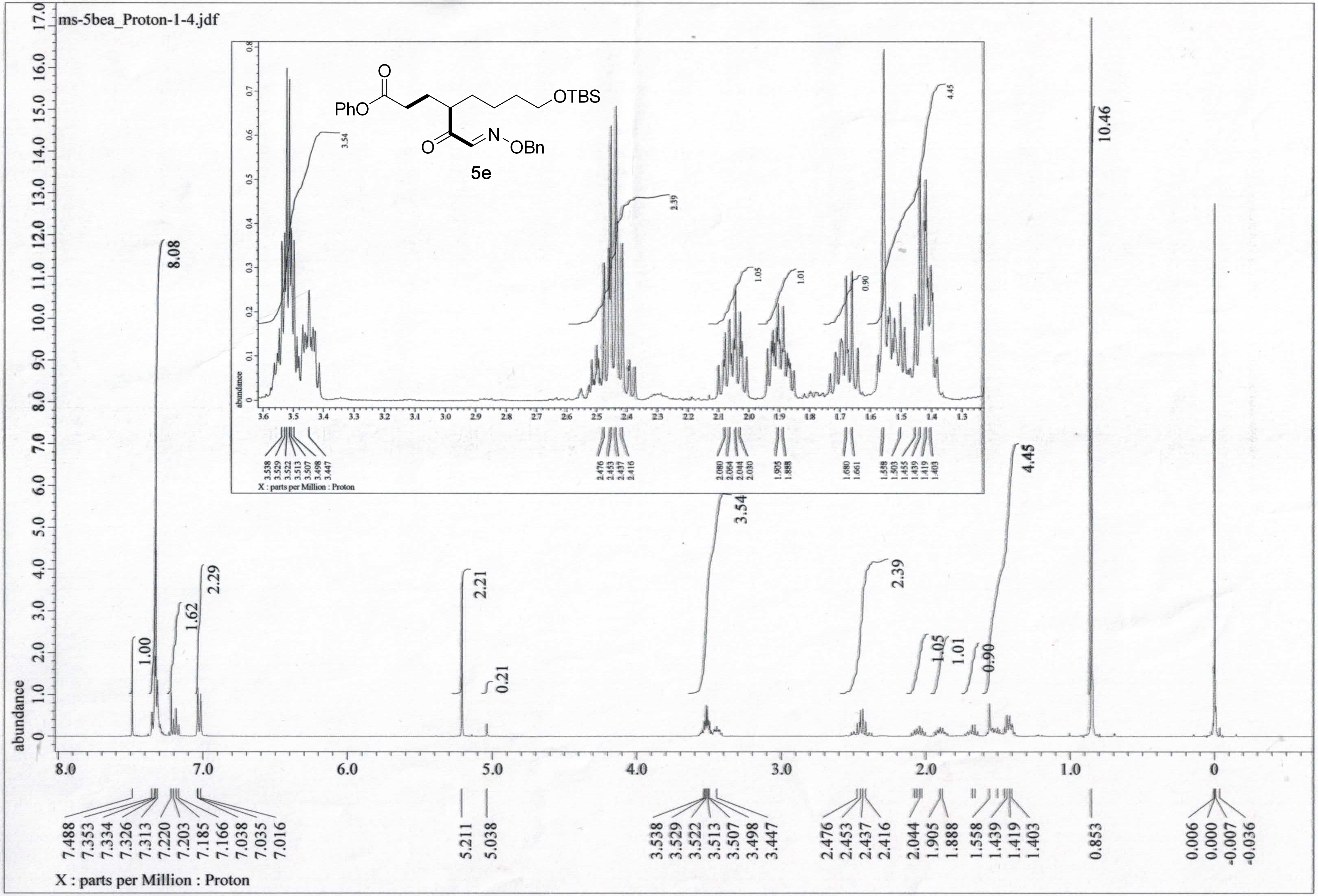

ms-5bea_Proton-1-4.jdf
PhO
O
OTBS
O
N
OBn
5e
abundance
X : parts per Million : Proton
8.08
1.00
1.62
2.29
2.21
0.21
3.54
2.39
1.05
1.01
0.90
4.45
10.46
7.488
7.353
7.334
7.326
7.313
7.220
7.203
7.185
7.166
7.038
7.035
7.016
5.211
5.038
3.538
3.529
3.522
3.513
3.507
3.498
3.447
2.476
2.453
2.437
2.416
2.080
2.064
2.044
2.030
1.905
1.888
1.680
1.661
1.558
1.503
1.455
1.439
1.419
1.403
0.853
0.006
0.000
-0.007
-0.036

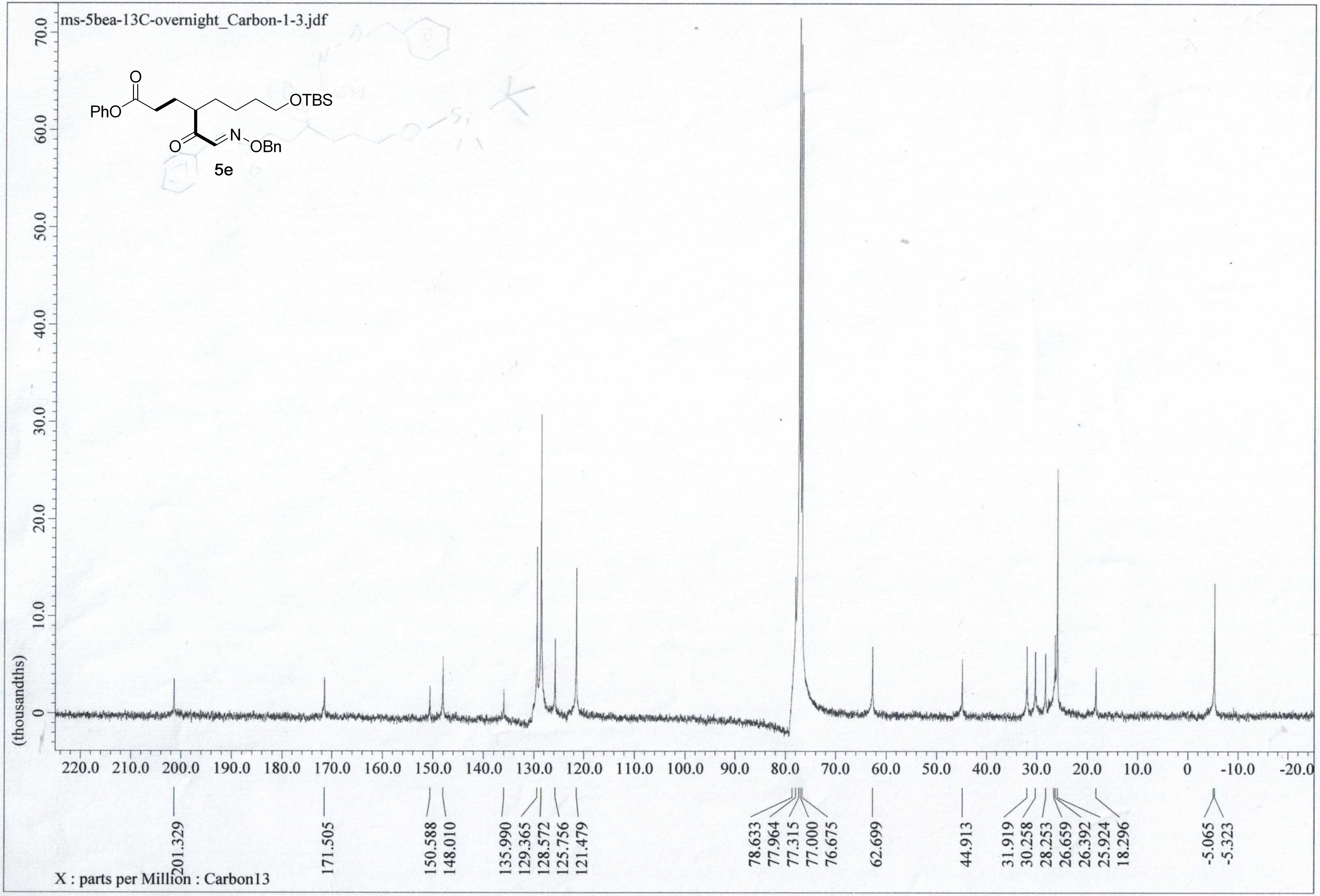
ms-5bea-13C-overnight_Carbon-1-3.jdf
PhO
O
OTBS
O
N
OBn
5e
(thousandths)
70.0
60.0
50.0
40.0
30.0
20.0
10.0
0
220.0 210.0 200.0 190.0 180.0 170.0 160.0 150.0 140.0 130.0 120.0 110.0 100.0 90.0 80.0 70.0 60.0 50.0 40.0 30.0 20.0 10.0 0 -10.0 -20.0
201.329
171.505
150.588
148.010
135.990
129.365
128.572
125.756
121.479
78.633
77.964
77.315
77.000
76.675
62.699
44.913
31.919
30.258
28.253
26.659
26.392
25.924
18.296
-5.065
-5.323
X : parts per Million : Carbon13

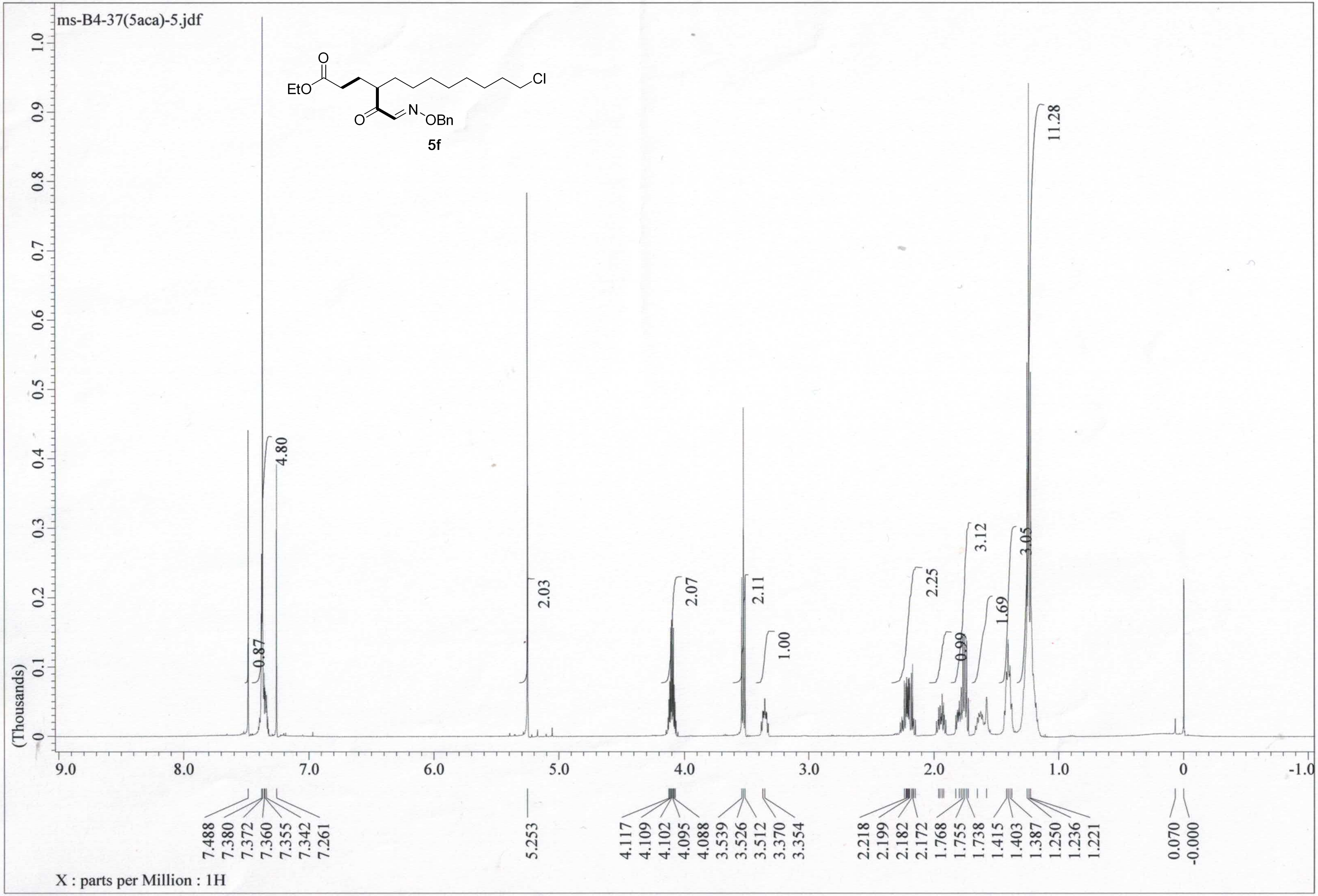
ms-B4-37(5aca)-5.jdf
EtO
O
O
N
OBn
Cl
5f
(Thousands)
X : parts per Million : 1H
0.87
4.80
2.03
2.07
2.11
1.00
2.25
0.99
3.12
1.69
3.05
11.28
7.488
7.380
7.372
7.360
7.355
7.342
7.261
5.253
4.117
4.109
4.102
4.095
4.088
3.539
3.526
3.512
3.370
3.354
2.218
2.199
2.182
2.172
1.768
1.755
1.738
1.415
1.403
1.387
1.250
1.236
1.221
0.070
-0.000

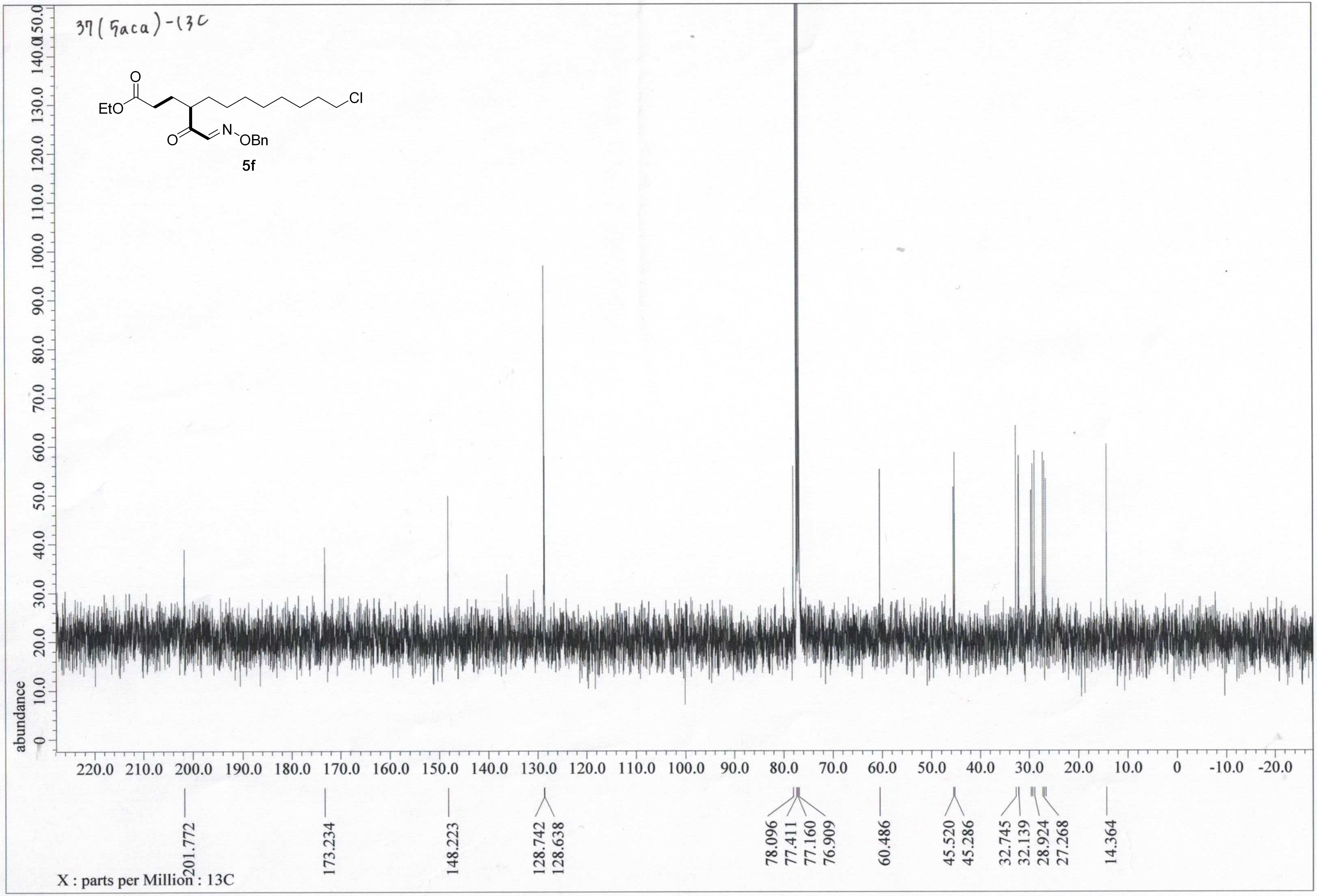
37(5aca)-13C
EtO
O
Cl
N
OBn
5f
abundance
X : parts per Million : 13C
201.772
173.234
148.223
128.742
128.638
78.096
77.411
77.160
76.909
60.486
45.520
45.286
32.745
32.139
28.924
27.268
14.364

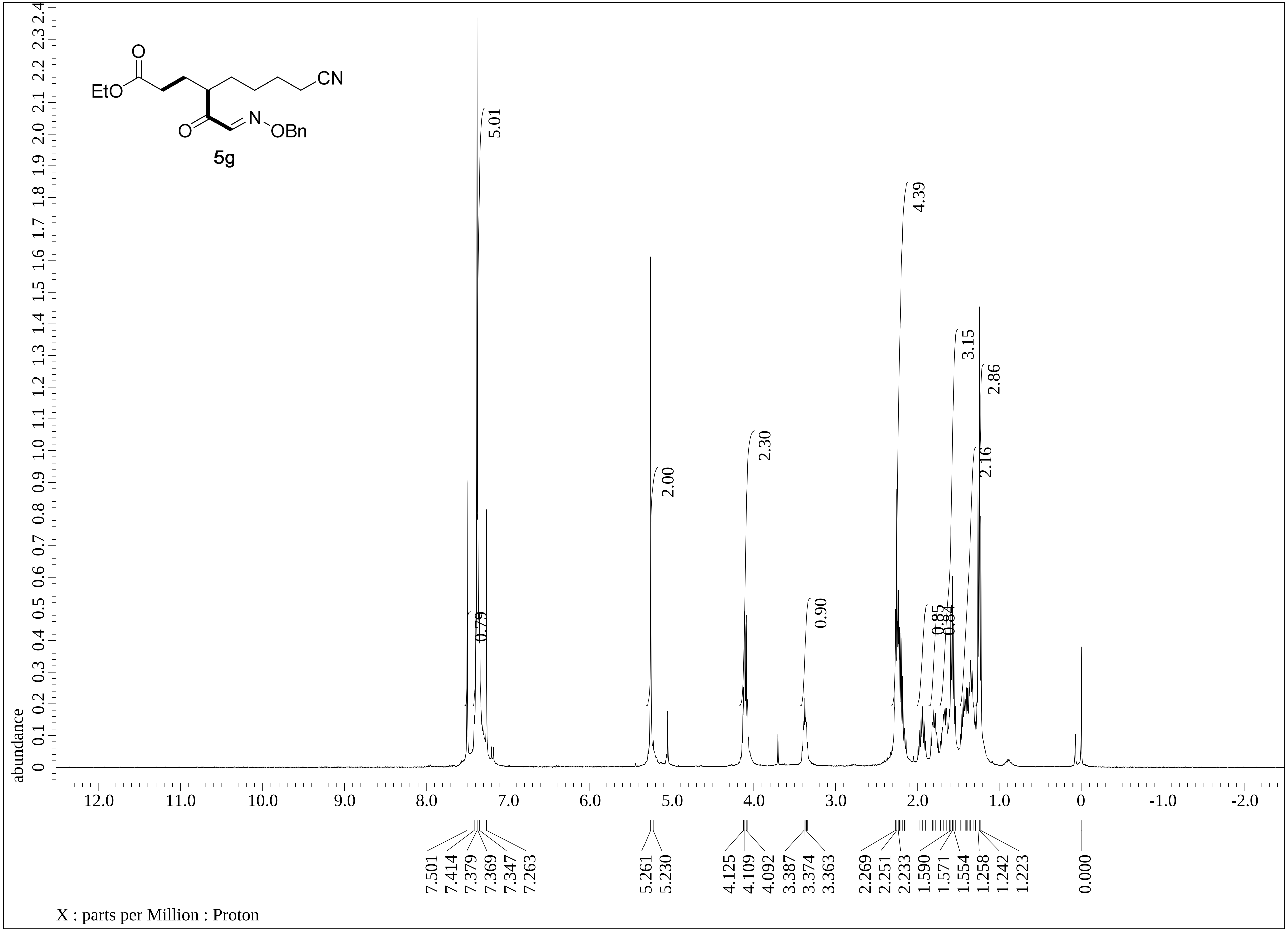
EtO
O
CN
N
OBn
O
5g
abundance
5.01
4.39
3.15
2.86
2.30
2.16
2.00
0.90
0.79
0.85
0.84
12.0
11.0
10.0
9.0
8.0
7.0
6.0
5.0
4.0
3.0
2.0
1.0
0
-1.0
-2.0
7.501
7.414
7.379
7.369
7.347
7.263
5.261
5.230
4.125
4.109
4.092
3.387
3.374
3.363
2.269
2.251
2.233
1.590
1.571
1.554
1.258
1.242
1.223
0.000
X : parts per Million : Proton

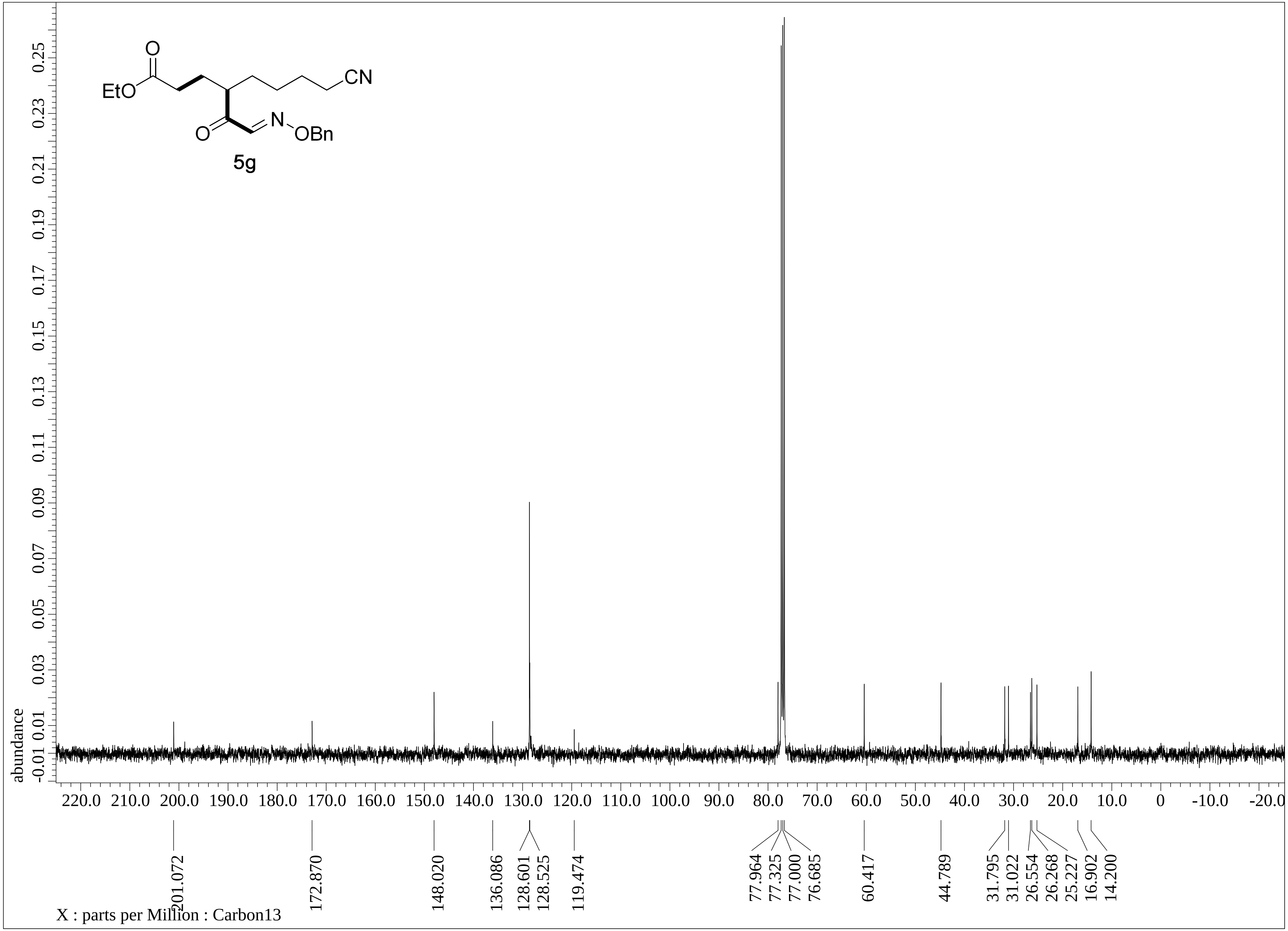
EtO
O
O
N
OBn
CN
5g
abundance
-0.01 0.01 0.03 0.05 0.07 0.09 0.11 0.13 0.15 0.17 0.19 0.21 0.23 0.25
220.0 210.0 200.0 190.0 180.0 170.0 160.0 150.0 140.0 130.0 120.0 110.0 100.0 90.0 80.0 70.0 60.0 50.0 40.0 30.0 20.0 10.0 0 -10.0 -20.0
201.072
172.870
148.020
136.086
128.601
128.525
119.474
77.964
77.325
77.000
76.685
60.417
44.789
31.795
31.022
26.554
26.268
25.227
16.902
14.200
X : parts per Million : Carbon13

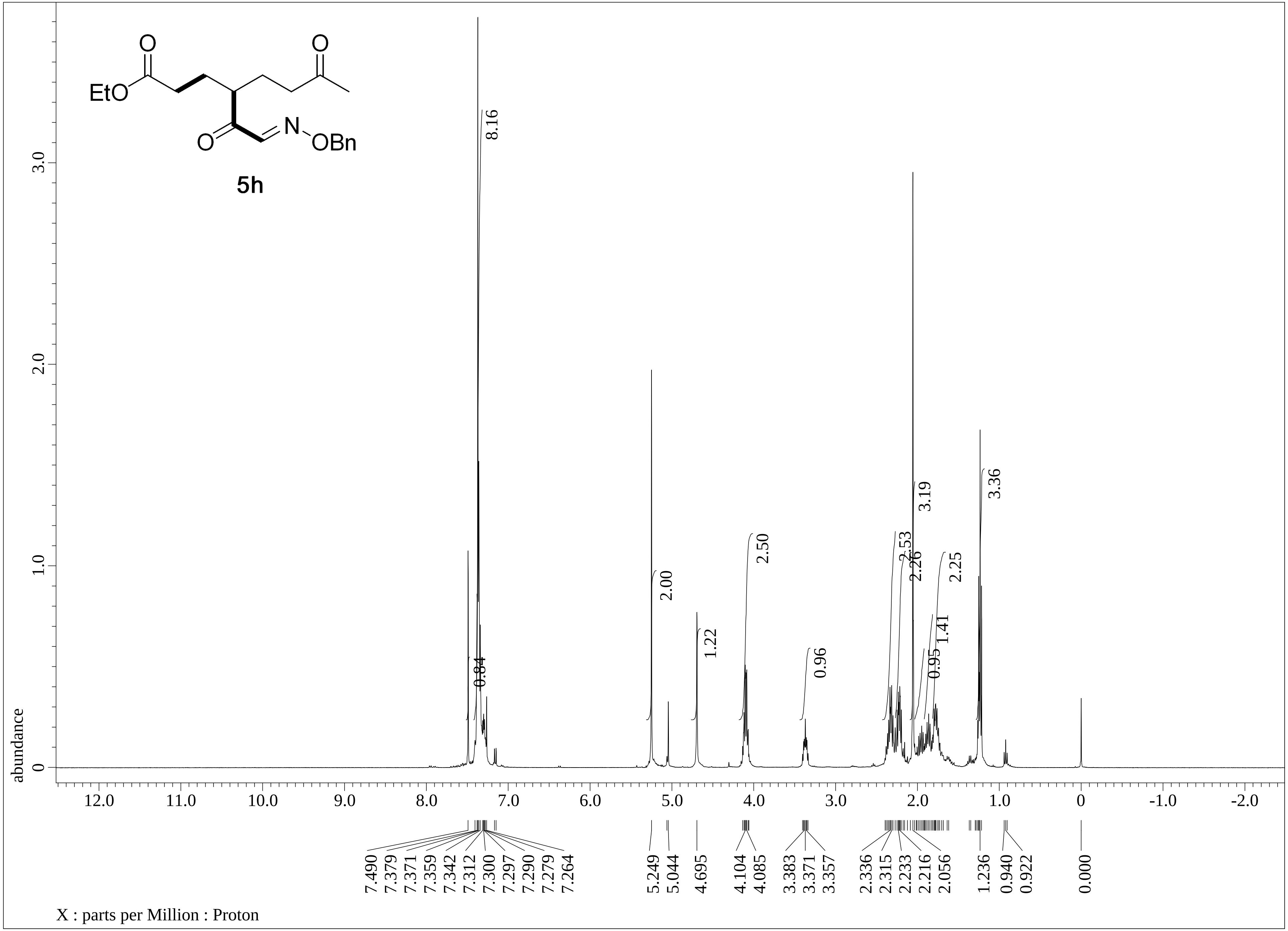

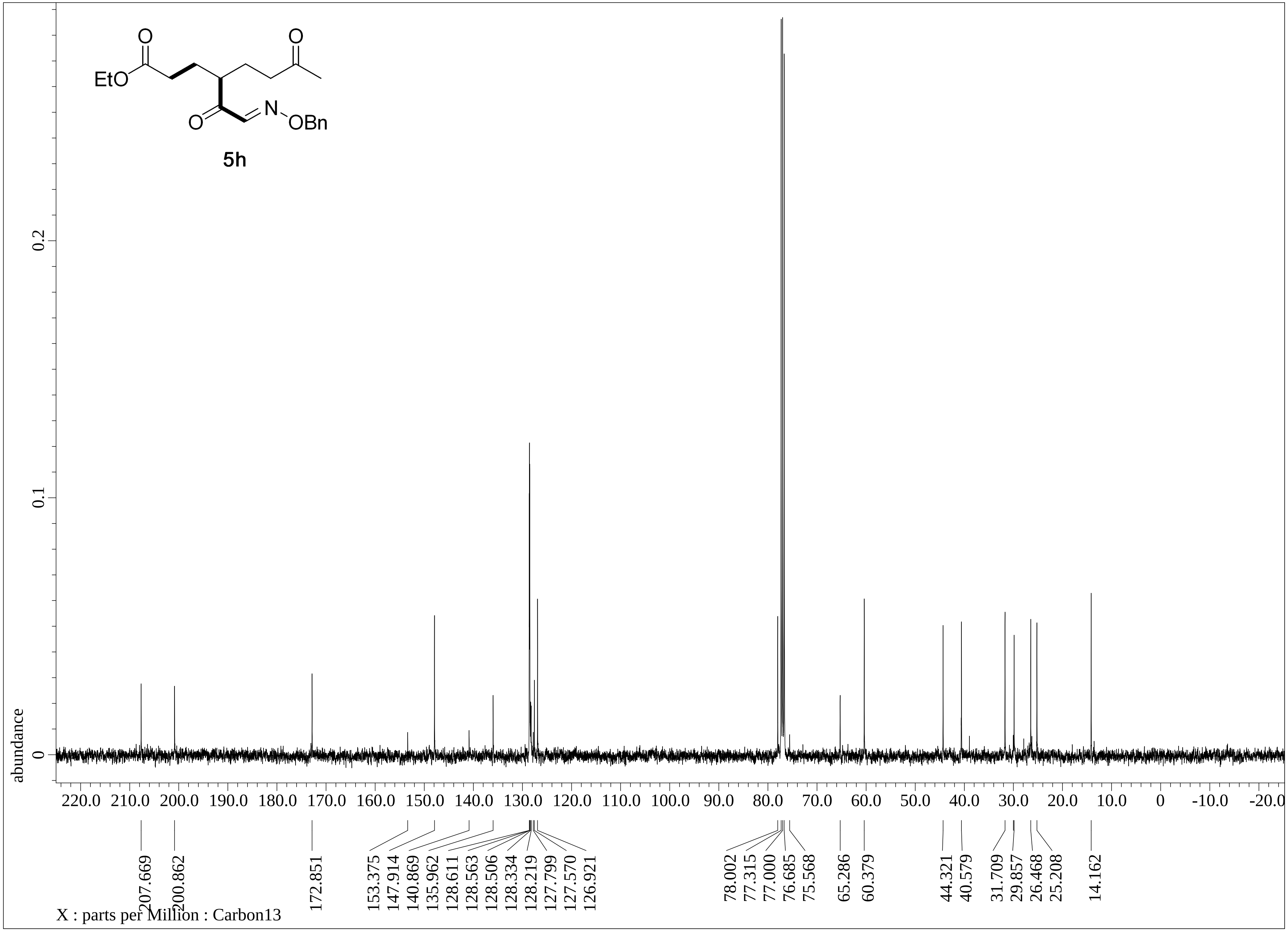
EtO
O
O
N
OBn
O
5h
abundance
0
0.1
0.2
220.0 210.0 200.0 190.0 180.0 170.0 160.0 150.0 140.0 130.0 120.0 110.0 100.0 90.0 80.0 70.0 60.0 50.0 40.0 30.0 20.0 10.0 0 -10.0 -20.0
207.669
200.862
172.851
153.375
147.914
140.869
135.962
128.611
128.563
128.506
128.334
128.219
127.799
127.570
126.921
78.002
77.315
77.000
76.685
75.568
65.286
60.379
44.321
40.579
31.709
29.857
26.468
25.208
14.162
X : parts per Million : Carbon13

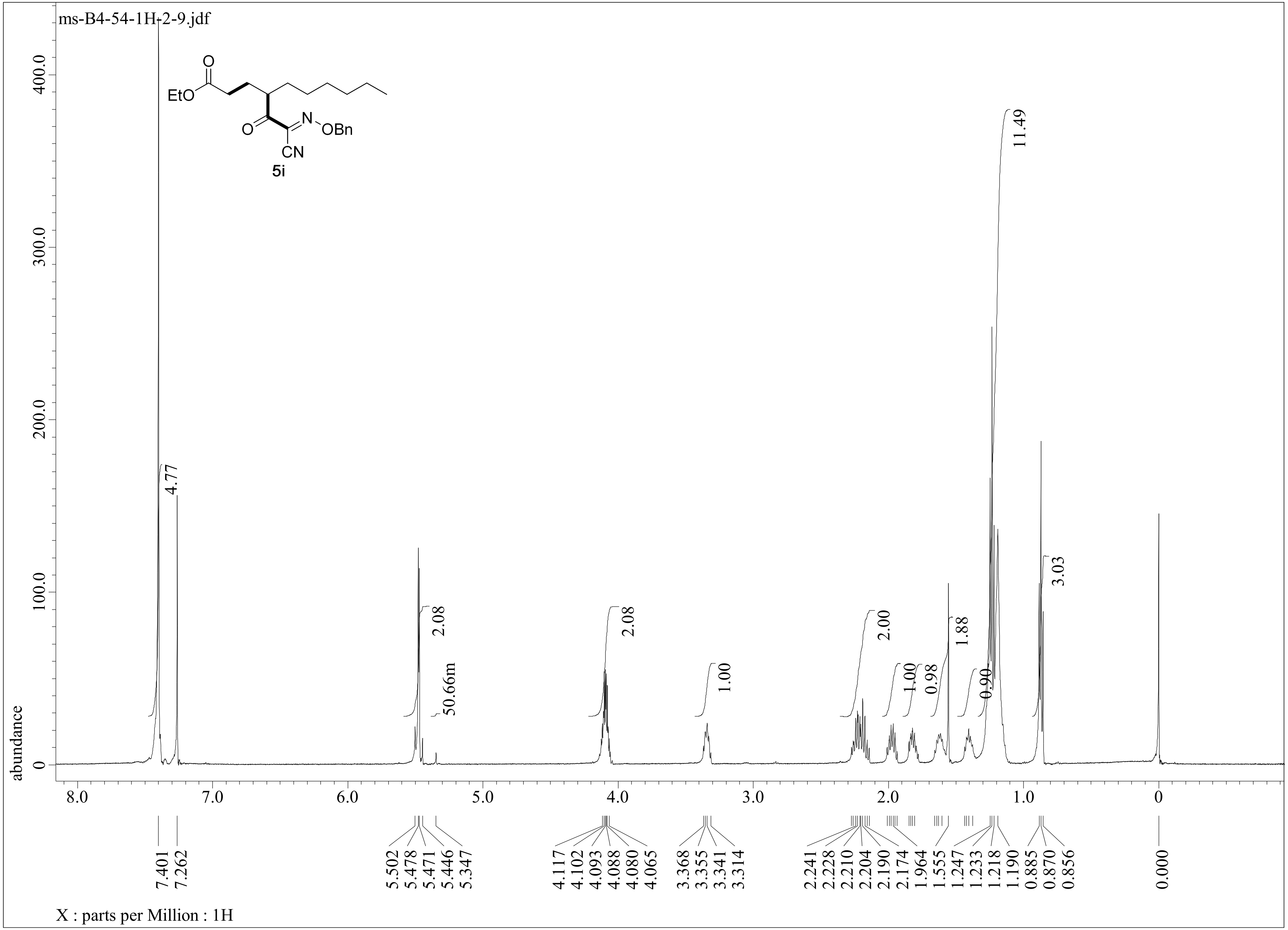
ms-B4-54-1H-2-9.jdf
EtO
O
O
N
OBn
CN
5i
abundance
400.0
300.0
200.0
100.0
0
4.77
2.08
50.66m
2.08
1.00
2.00
1.00
0.98
1.88
0.90
11.49
3.03
8.0
7.0
6.0
5.0
4.0
3.0
2.0
1.0
0
7.401
7.262
5.502
5.478
5.471
5.446
5.347
4.117
4.102
4.093
4.088
4.080
4.065
3.368
3.355
3.341
3.314
2.241
2.228
2.210
2.204
2.190
2.174
1.964
1.555
1.247
1.233
1.218
1.190
0.885
0.870
0.856
0.000
X : parts per Million : 1H

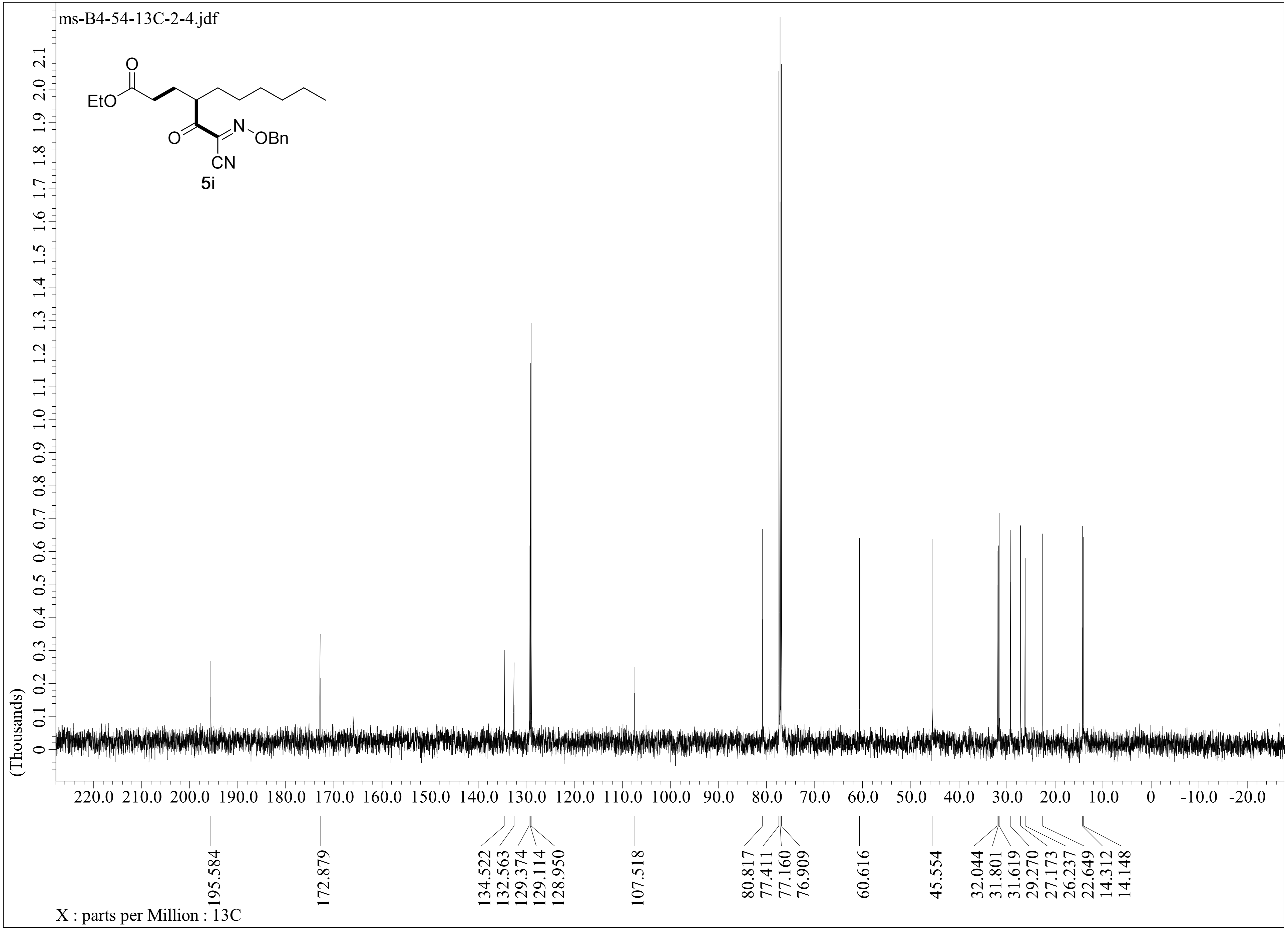
ms-B4-54-13C-2-4.jdf
EtO
O
O
N
OBn
CN
5i
(Thousands)
195.584
172.879
134.522
132.563
129.374
129.114
128.950
107.518
80.817
77.411
77.160
76.909
60.616
45.554
32.044
31.801
31.619
29.270
27.173
26.237
22.649
14.312
14.148
X : parts per Million : 13C

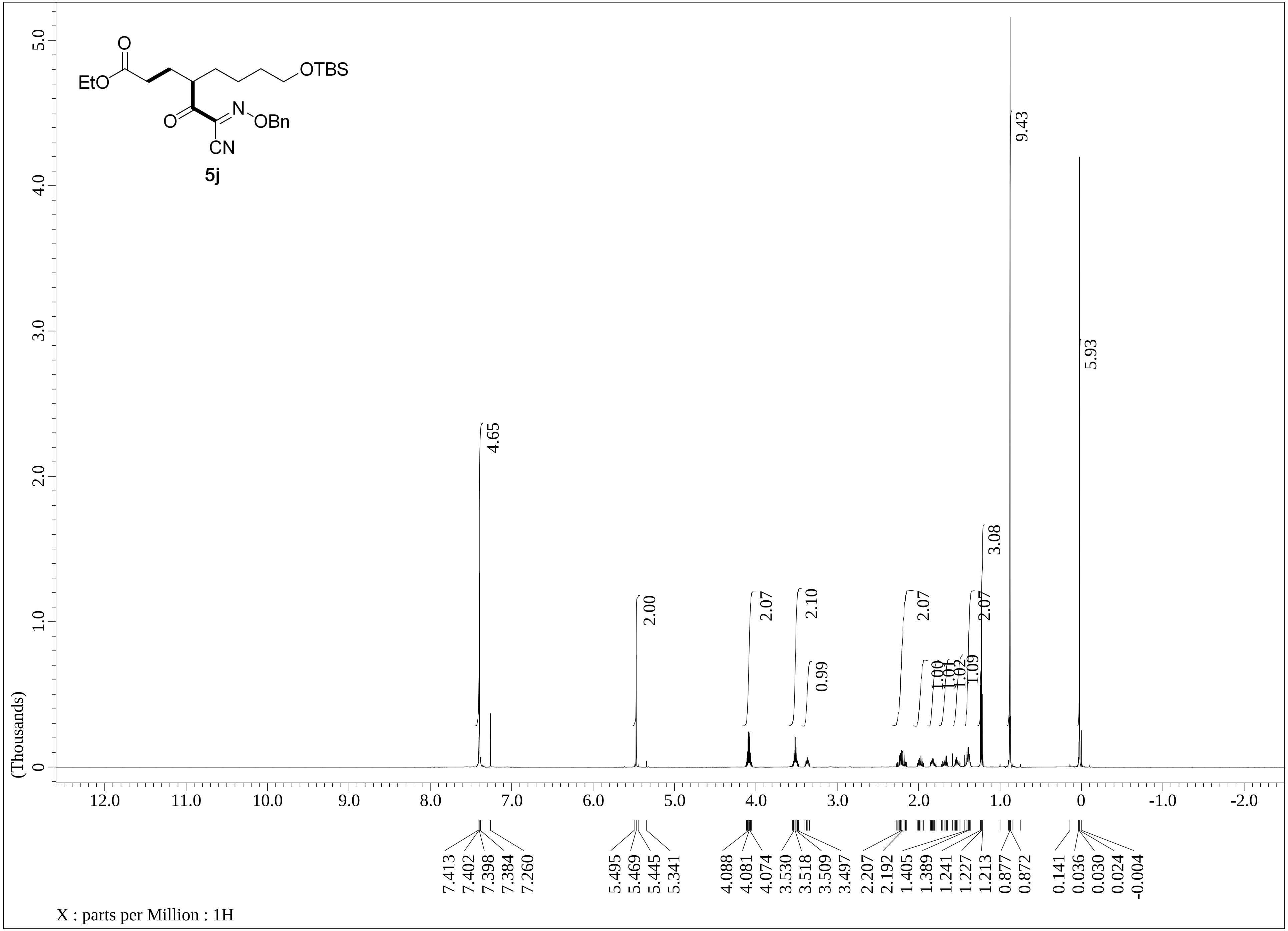

EtO
O
OTBS
O
N
OBn
CN
5j
5.0
4.0
3.0
2.0
1.0
0
(Thousands)
4.65
2.00
2.07
2.10
0.99
2.07
1.00
1.01
1.05
1.02
1.09
2.07
3.08
9.43
5.93
12.0
11.0
10.0
9.0
8.0
7.0
6.0
5.0
4.0
3.0
2.0
1.0
0
-1.0
-2.0
7.413
7.402
7.398
7.384
7.260
5.495
5.469
5.445
5.341
4.088
4.081
4.074
3.530
3.518
3.509
3.497
2.207
2.192
1.405
1.389
1.241
1.227
1.213
0.877
0.872
0.141
0.036
0.030
0.024
-0.004
X : parts per Million : 1H

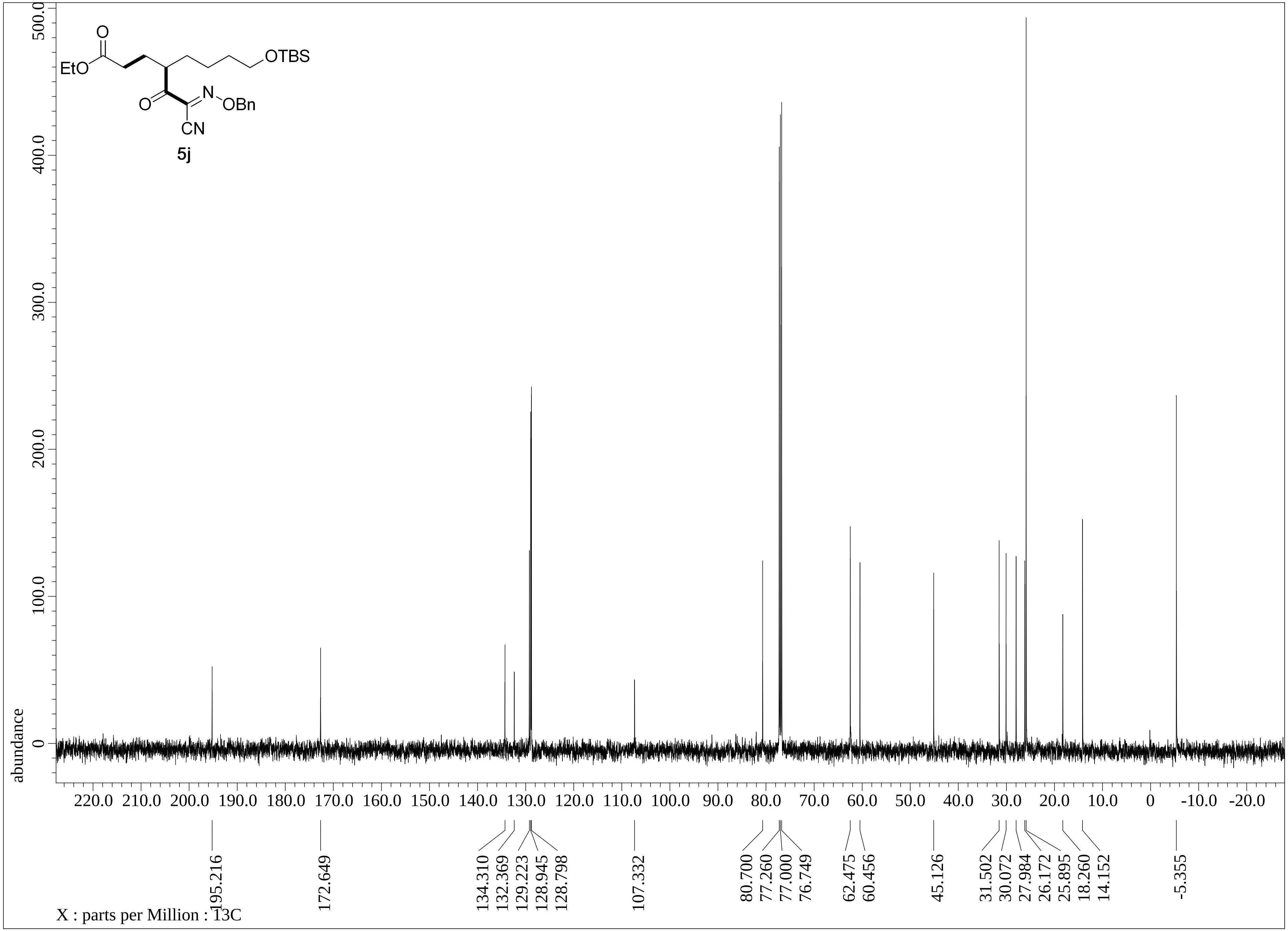

EtO
O
OTBS
N
OBn
O
CN
5j
abundance
500.0
400.0
300.0
200.0
100.0
0
220.0 210.0 200.0 190.0 180.0 170.0 160.0 150.0 140.0 130.0 120.0 110.0 100.0 90.0 80.0 70.0 60.0 50.0 40.0 30.0 20.0 10.0 0 -10.0 -20.0
195.216
172.649
134.310
132.369
129.223
128.945
128.798
107.332
80.700
77.260
77.000
76.749
62.475
60.456
45.126
31.502
30.072
27.984
26.172
25.895
18.260
14.152
-5.355
X : parts per Million : 13C

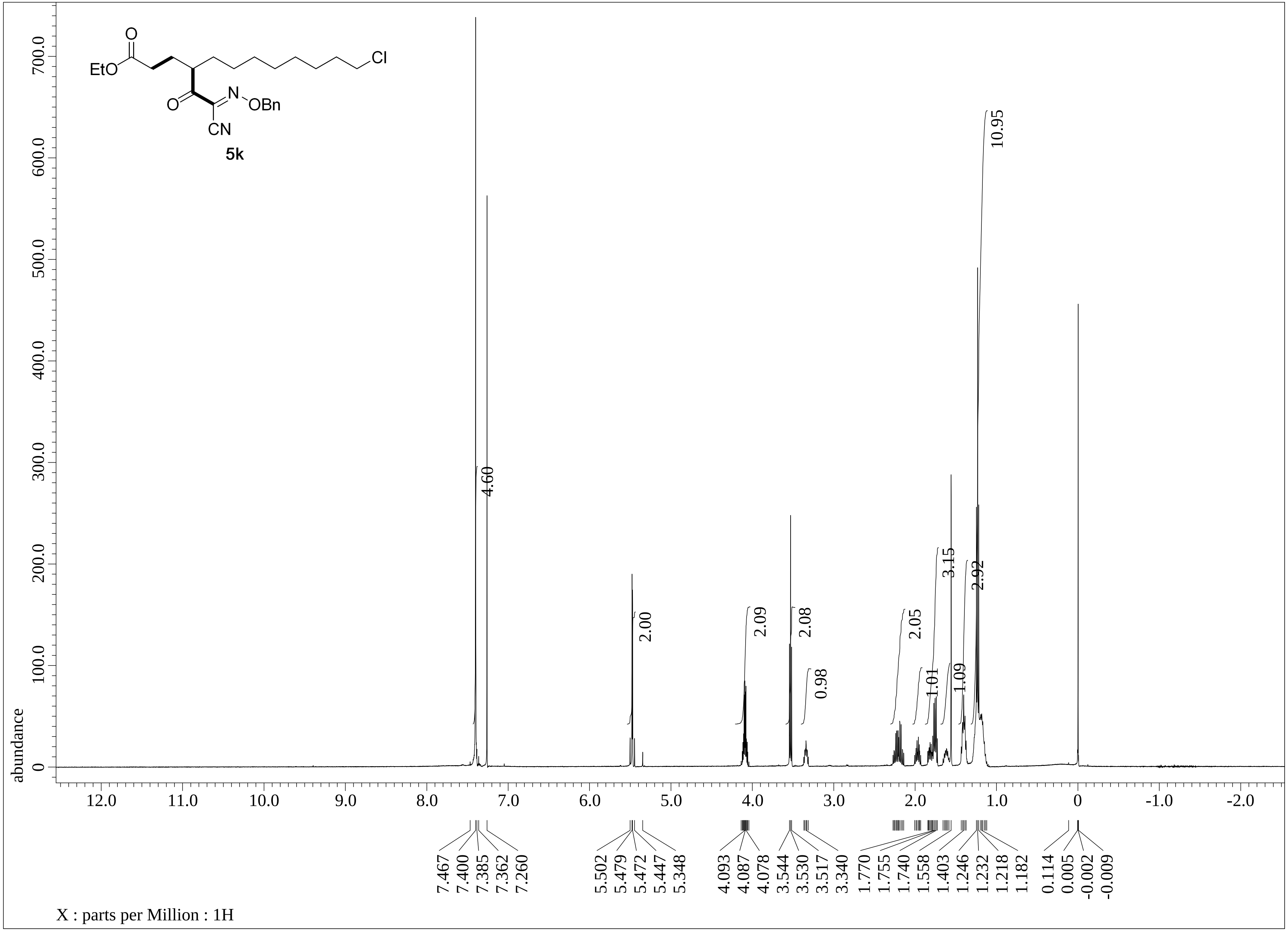

EtO
O
Cl
O
N
OBn
CN
5k
abundance
700.0
600.0
500.0
400.0
300.0
200.0
100.0
0
4.60
2.00
2.09
2.08
0.98
2.05
1.01
3.15
1.09
2.92
10.95
12.0
11.0
10.0
9.0
8.0
7.0
6.0
5.0
4.0
3.0
2.0
1.0
0
-1.0
-2.0
7.467
7.400
7.385
7.362
7.260
5.502
5.479
5.472
5.447
5.348
4.093
4.087
4.078
3.544
3.530
3.517
3.340
1.770
1.755
1.740
1.558
1.403
1.246
1.232
1.218
1.182
0.114
0.005
-0.002
-0.009
X : parts per Million : 1H

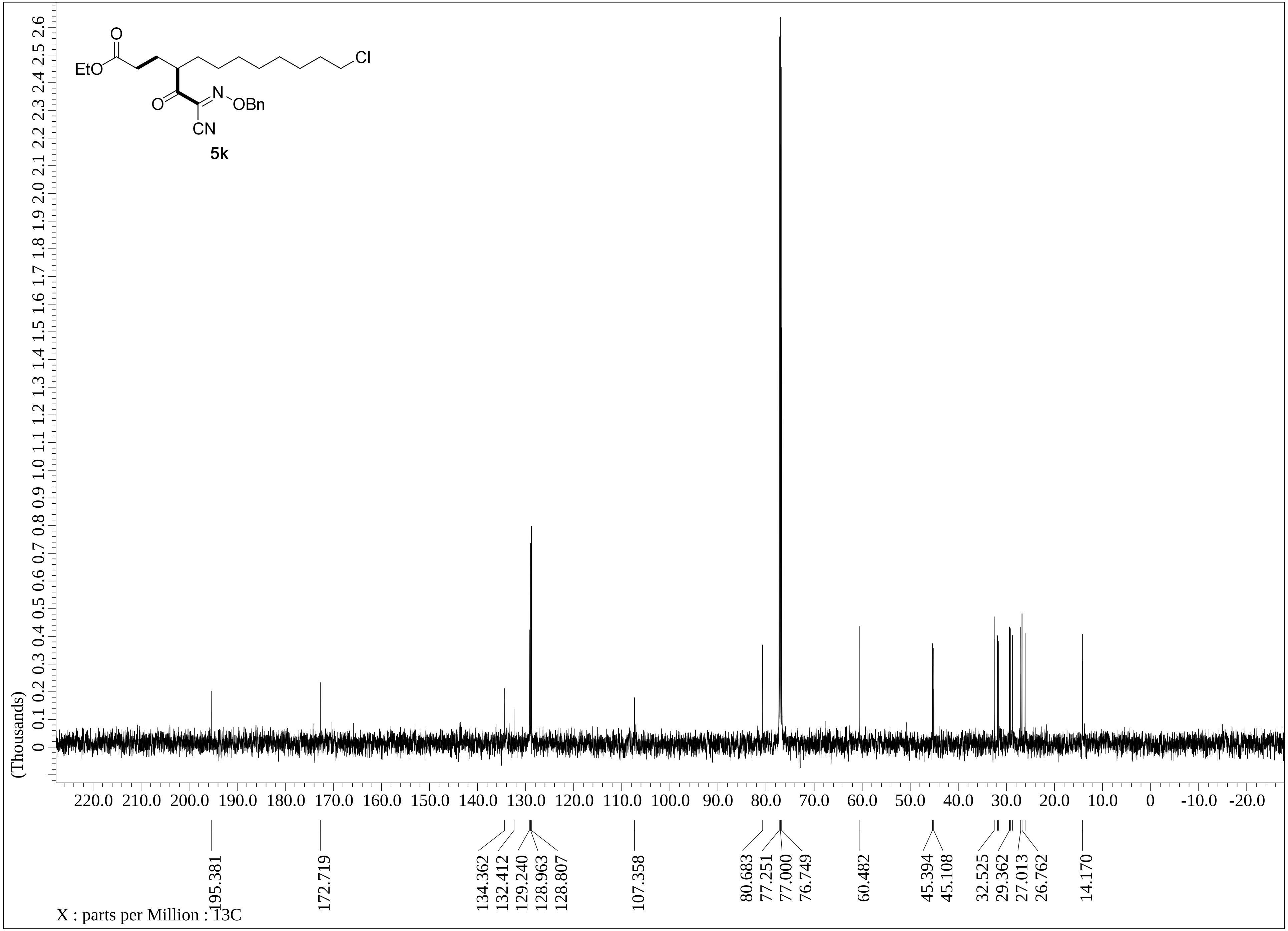

EtO
O
Cl
O
N
OBn
CN
5k
(Thousands)
0 0.1 0.2 0.3 0.4 0.5 0.6 0.7 0.8 0.9 1.0 1.1 1.2 1.3 1.4 1.5 1.6 1.7 1.8 1.9 2.0 2.1 2.2 2.3 2.4 2.5 2.6
220.0 210.0 200.0 190.0 180.0 170.0 160.0 150.0 140.0 130.0 120.0 110.0 100.0 90.0 80.0 70.0 60.0 50.0 40.0 30.0 20.0 10.0 0 -10.0 -20.0
195.381
172.719
134.362
132.412
129.240
128.963
128.807
107.358
80.683
77.251
77.000
76.749
60.482
45.394
45.108
32.525
29.362
27.013
26.762
14.170
X : parts per Million : 13C

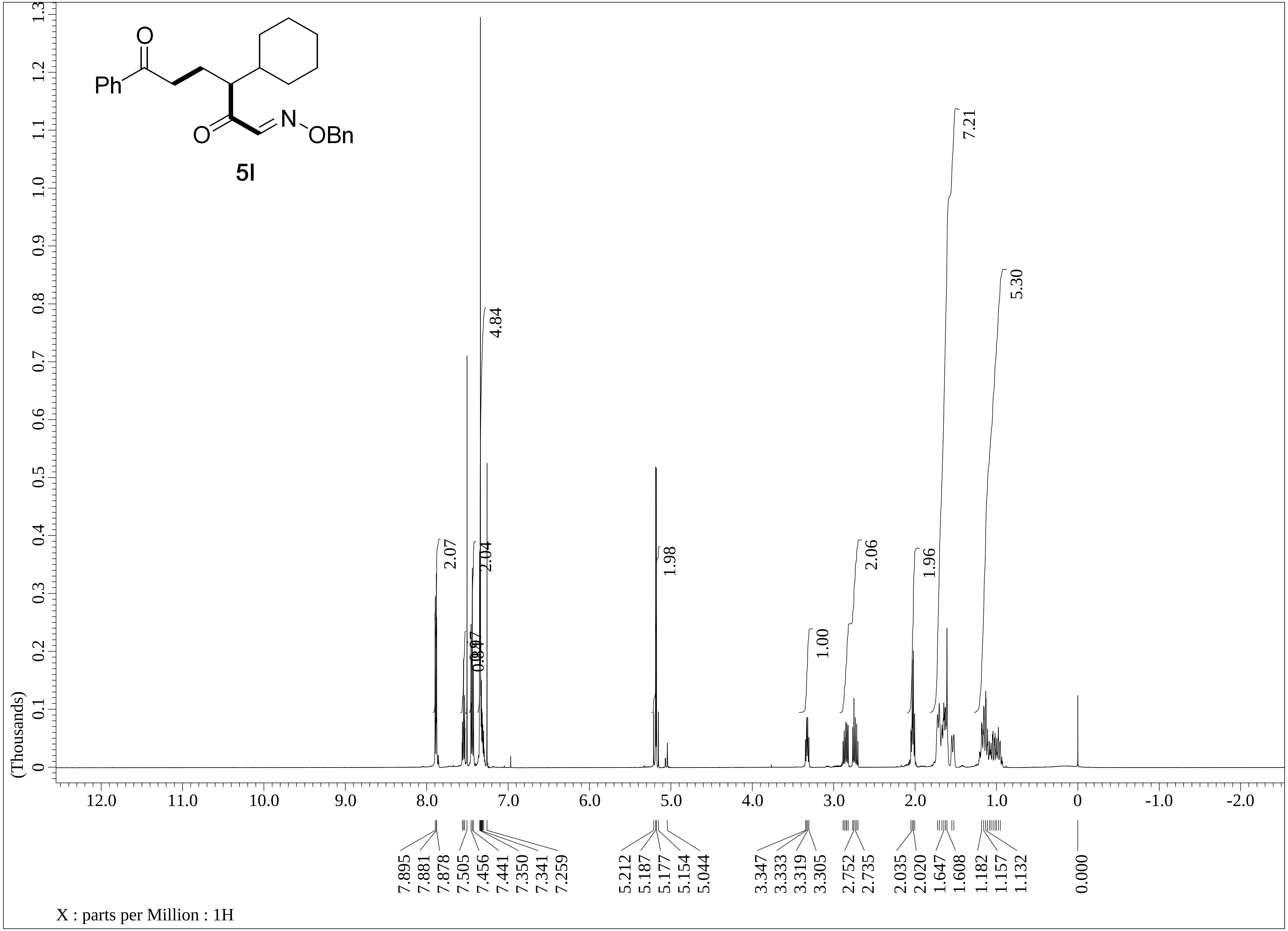

O
Ph
N
O
OBn
5l
1.3
1.2
1.1
1.0
0.9
0.8
0.7
0.6
0.5
0.4
0.3
0.2
0.1
0
(Thousands)
2.07
2.04
4.84
1.98
1.00
2.06
1.96
7.21
5.30
12.0
11.0
10.0
9.0
8.0
7.0
6.0
5.0
4.0
3.0
2.0
1.0
0
-1.0
-2.0
7.895
7.881
7.878
7.505
7.456
7.441
7.350
7.341
7.259
5.212
5.187
5.177
5.154
5.044
3.347
3.333
3.319
3.305
2.752
2.735
2.035
2.020
1.647
1.608
1.182
1.157
1.132
0.000
X : parts per Million : 1H

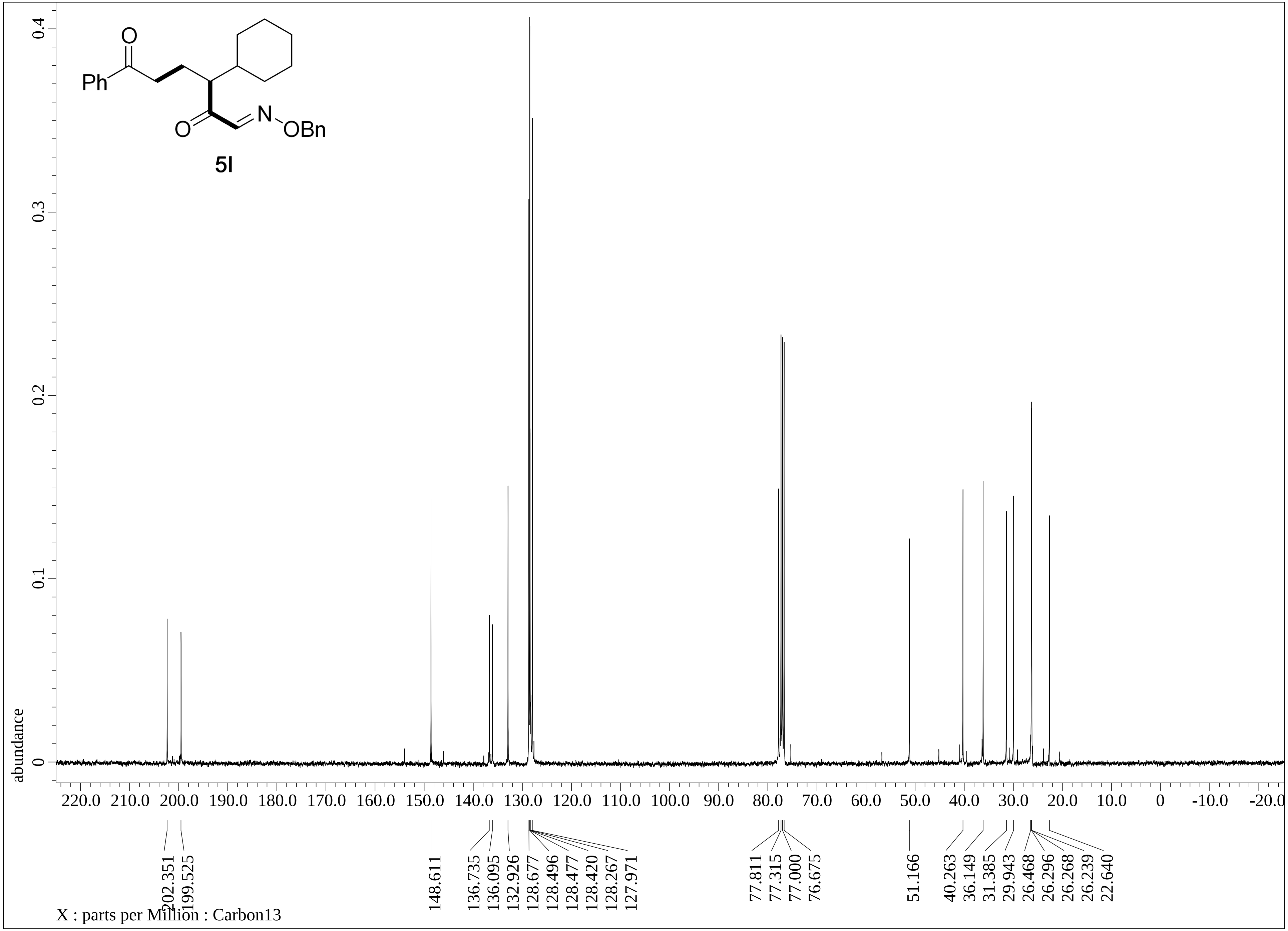

O
Ph
N
O
OBn
5l
0.4
0.3
0.2
0.1
0
abundance
220.0 210.0 200.0 190.0 180.0 170.0 160.0 150.0 140.0 130.0 120.0 110.0 100.0 90.0 80.0 70.0 60.0 50.0 40.0 30.0 20.0 10.0 0 -10.0 -20.0
202.351
199.525
148.611
136.735
136.095
132.926
128.677
128.496
128.477
128.420
128.267
127.971
77.811
77.315
77.000
76.675
51.166
40.263
36.149
31.385
29.943
26.468
26.296
26.268
26.239
22.640
X : parts per Million : Carbon13
